# Nanovaccine Delivery Approaches and Advanced Delivery Systems for the Prevention of Viral Infections: From Development to Clinical Application

**DOI:** 10.3390/pharmaceutics13122091

**Published:** 2021-12-05

**Authors:** Ana Sara Cordeiro, Yogita Patil-Sen, Maitreyi Shivkumar, Ronak Patel, Abdulwahhab Khedr, Mohamed A. Elsawy

**Affiliations:** 1 Leicester Institute for Pharmaceutical Innovation, Leicester School of Pharmacy, De Montfort University, Leicester LE1 9BH, UK; sara.cordeiro@dmu.ac.uk (A.S.C.); maitreyi.shivkumar@dmu.ac.uk (M.S.); p2577396@alumni365.dmu.ac.uk (A.K.); 2 Wrightington, Wigan and Leigh Teaching Hospitals NHS Foundation Trust, National Health Service, Wigan WN6 0SZ, UK; Yogita.Patil-Sen@wwl.nhs.uk; 3 School of Pharmacy and Biomedical Sciences, University of Central Lancashire, Preston PR1 2HE, UK; patel_ronak94@hotmail.com; 4 Department of Pharmaceutics and Industrial Pharmacy, Faculty of Pharmacy, Zagazig University, Zagazig 44519, Egypt

**Keywords:** vaccine, virus, nanomaterials, delivery, immunisation

## Abstract

Viral infections causing pandemics and chronic diseases are the main culprits implicated in devastating global clinical and socioeconomic impacts, as clearly manifested during the current COVID-19 pandemic. Immunoprophylaxis via mass immunisation with vaccines has been shown to be an efficient strategy to control such viral infections, with the successful and recently accelerated development of different types of vaccines, thanks to the advanced biotechnological techniques involved in the upstream and downstream processing of these products. However, there is still much work to be done for the improvement of efficacy and safety when it comes to the choice of delivery systems, formulations, dosage form and route of administration, which are not only crucial for immunisation effectiveness, but also for vaccine stability, dose frequency, patient convenience and logistics for mass immunisation. In this review, we discuss the main vaccine delivery systems and associated challenges, as well as the recent success in developing nanomaterials-based and advanced delivery systems to tackle these challenges. Manufacturing and regulatory requirements for the development of these systems for successful clinical and marketing authorisation were also considered. Here, we comprehensively review nanovaccines from development to clinical application, which will be relevant to vaccine developers, regulators, and clinicians.

## 1. Introduction

Viral infections can have substantial negative clinical and socioeconomic impact globally [1,2,3]. Recently, the COVID-19 pandemic (caused by SARS-CoV-2 virus) brought about devastating clinical effects, with more than 186 million confirmed cases globally and ~5 million deaths reported by the WHO so far [4]. The socioeconomic impact was as bad: it is estimated that losses to the global economy amounted to £8 trillion over 2020–21 (with a global GDP loss of 6.7%), and will reach £22 trillion over 2020–25 [5,6]. It is also estimated that seasonal influenza can cause 250,000–500,000 annual deaths worldwide [1].

Chronic diseases caused by viral infections can be just as impactful; for instance, the number of known HIV infections since AIDS was first diagnosed is 77.5 million, with 37.7 million total deaths and 1.5 million new infections in 2020 [7]. Another example is hepatitis C, of which 71 million infections were reported worldwide, with more than one in ten cases resulting in severe liver cirrhosis [8]. These are just few examples of many, where the increased number of infections with various viruses place an extreme burden on healthcare sectors globally, resulting in the hospitalisation of high-risk patients, contributing to significant increases in healthcare expenditures and diminishing the ability of current infrastructure to meet the clinical needs of all patients [9,10].

Immunoprophylaxis through mass immunisation with vaccines has proven to be a successful strategy to control viral infections and contain the catastrophic impacts of pandemics. Vaccines can be broadly divided into whole virus, subunit, or genetic. Whole virus approaches such as live attenuated or inactivated (killed) vaccines are the oldest technology. Live attenuated vaccines, such as the influenza (FluMist nasal spray), varicella (chicken pox), oral rotavirus, oral polio (OPV), MMR, and Yellow Fever vaccines contain a weakened form of the virus. This triggers an immune response without causing disease. Attenuation can be achieved by serial passaging in a foreign host, or genetic modification of the virulent genes. Although these vaccines induce strong immune responses, risk of infection in immunocompromised individuals or potential mutations that revert to more pathogenic forms need to be considered. For instance, cases of vaccine-associated paralytic poliomyelitis due to the genetic instability of the Sabin OPV have been reported [11,12], leading to the development of novel OPV vaccines with enhanced stability to mitigate reversion to neurovirulence [13]. Mild local immune reactions at the site of inoculation have been reported for several live attenuated vaccines, although severe adverse reactions are rare [14]; for example, anaphylaxis, i.e., febrile seizures, occurred in 0.001–0.03% of children who received the MMR vaccine, and vaccine-induced measles has been reported in severely immunocompromised individuals [15], while the yellow fever vaccine can induce possibly fatal vaccine-associated viscerotropic disease [16]. Inactivated vaccines, on the other hand, are nonreplicating, and consist of whole viruses which have been inactivated by heat, UV radiation, or fixation. Examples include the Salk inactivated polio vaccine (IPV), and more recently, the SARS-CoV-2 vaccines CoronaVac, created by Sinovac (licensed in China) and Bharat Biotech’s Covaxin, licensed in India [17]. Inactivated vaccines are safer than live attenuated vaccines, although the induced responses can be weaker. This requires higher amounts of the vaccine to be administered, and often with an adjuvant. Despite this, seroconversion has been observed in individuals vaccinated with CoronaVac, with neutralizing antibodies supporting protection against COVID-19 [18,19,20].

Protein subunit vaccines consist of recombinant proteins or protein fragments, typically with highly immunogenic antigens that induce an immune response [21]. For instance, human papillomavirus (HPV) vaccines comprise the HPV L1 capsid protein in virus-like particles (VLP), derived from multiple genotypes that are most commonly associated with cervical cancers [22]. It has been suggested that the high density of the repetitive antigens may induce high titres of neutralising antibodies that contributes to the exceptional potency of this vaccine [23]. Subunit vaccines are generally administered with an adjuvant and have no risk of causing disease. Multiple subunit vaccines against SARS-CoV-2, providing recombinant spike or spike fragments, are currently in development [24].

The genetic approach of DNA or mRNA vaccines is by far the most novel [25]. Viral DNA or mRNA delivered directly to host cell induces the production of the coded viral proteins, which, in turn, activates the immune system. The injection of DNA was shown to elicit immune responses and offer protection against viral infections as early as the 1990s [26,27], and has since been evaluated against both viral and nonviral diseases in various clinical trials [28]. Similarly, various mRNA vaccine platforms have been developed within the last decade; however, their utility has been limited until recently due to the instability of mRNA and inefficient delivery [29]. The urgency brought about by the COVID-19 pandemic, combined with the speed and relative ease of mRNA synthesis, allowed the rapid development and validation of mRNA vaccines. Both the BNT162 Pfizer/BioNTech and Moderna mRNA vaccines have shown nearly 95% efficacy against COVID-19 [30,31]. In addition to the low cost of production, mRNA vaccines can also be adapted rapidly against new variants or novel viruses, suggesting promising alternatives to more conventional vaccine approaches.

The development of these different types of vaccines against viruses has proven challenging over the years, but successful, thanks to the fast progress and recent advances in the development of the biotechnological techniques involved in the upstream and downstream processing of vaccines. However, there is still much work to be done to improve the efficacy and safety of the developed vaccines in order to gain a greater control over viruses. Besides challenges of dose reduction, side effects, cost-effectiveness and manufacturing capacity, the choices of dosage form and route of administration are crucial factors to consider when developing new vaccine products, as these will not only affect immunisation effectiveness, but also vaccine stability, dose frequency, patient convenience and logistics for mass immunisation. In this review, we will shed light on the main challenges associated with vaccine delivery and the recent success in developing nanomaterials-based, advanced delivery systems to overcome these challenges. We will also discuss the regulatory requirements for the development of these systems for successful clinical and marketing authorisation.

## 2. Challenges of Vaccine Delivery

With increasing knowledge of the immune system, researchers are beginning to apply vaccine formulations for the treatment of viral and other diseases, including cancer. As discussed above, different types of vaccines have been developed throughout the centuries, with different formulations and delivery requirements. Initially, most vaccines were composed of live attenuated or inactivated pathogens, eliciting an immune response through the same pathways that would be in place to fight the original disease. However, these types of vaccines encompass a risk of severe side effects, which led to the development of recombinant antigens with better safety profiles. These proteins, peptides and nucleic acid antigens generally lead to specific responses, albeit usually requiring the addition of an adjuvant system to the formulation to improve their immunogenicity [32].

The classical adjuvant used in vaccines, and the first one to be approved for human use, is alum, a generic name for aluminium salts including aluminium phosphate and hydroxide. The mechanism of action of this adjuvant has been widely discussed and was initially thought to be related to the formation of a depot at the injection site, from where the adsorbed antigen would slowly be released into the systemic circulation. More recently, other studies have proposed different mechanisms for alum’s adjuvant activity, including induction of chemokine and cytokine secretion for immune cell recruitment, induction of local cell death and activation of innate immunity signalling pathways, among others [33].

Despite its effectiveness, alum presents many limitations as an adjuvant, namely, its sensitivity to freezing and instability at high temperatures, which mean that vaccines containing this adjuvant must be stored and transported under strict “cold chain” conditions. This requirement is a major constraint to vaccine deployment in low-income countries, where resources to maintain cold chain storage and transport are often unavailable [34]. On the other hand, alum has also shown lower adjuvant efficacy with peptide antigens and limited ability to elicit cellular immune responses, as are often required for intracellular pathogens such as HIV [35,36].

Another important aspect to take into consideration is the stability of the antigen in the vaccine formulation. Modern antigens, particularly peptides, proteins and nucleic acids, are generally sensitive to temperature, pH and enzymatic degradation. For this reason, it is essential to formulate them in delivery systems that provide protection from these degradation agents, allowing efficient antigen delivery to antigen presenting cells (APCs). Nano and microparticulate carriers are especially well-suited to perform this function, with demonstrated evidence of their efficacy widely available in the literature [37,38].

Finally, another major challenge in vaccine delivery is related to antigen targeting of relevant tissues such as lymph nodes (LN) and other lymphatic tissues, with abundant immune cell populations [39]. Targeting depends not only on the administration route, but also on the physicochemical properties of the selected antigen delivery system. In particular, particle size, surface charge and chemical modifications of the carriers’ surface have been shown to significantly influence their trafficking from the administration site to the relevant tissues and their interaction with relevant immune cell populations, particularly APCs [40,41,42]. Therefore, it is crucial that these aspects are considered in the development of novel vaccines against infectious viruses.

## 3. Routes of Vaccines Administration

**Figure 1:**
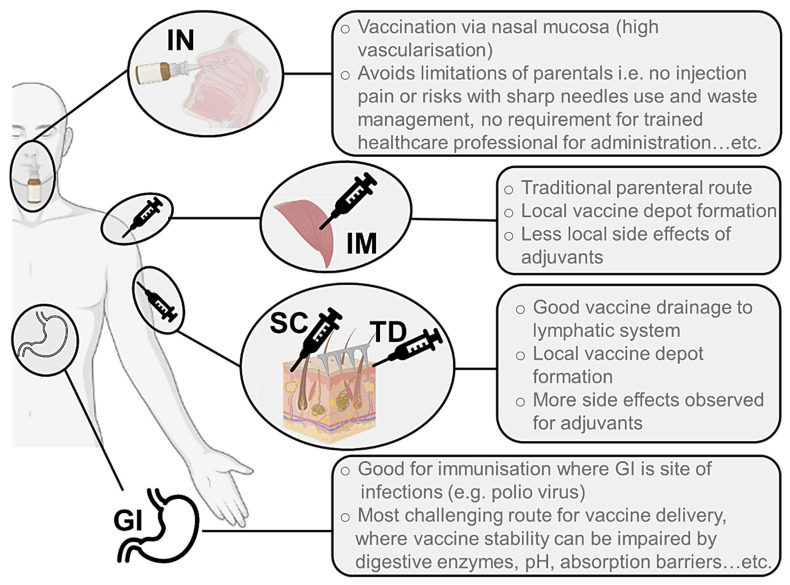
Illustration of the main routes of administration used for delivery of vaccines against viruses. These are IN (intranasal), IM (intramuscular), SC (subcutaneous), TD (transdermal) and oral (GI, gastrointestinal).

Vaccines have traditionally been delivered through parenteral routes, particularly through the intramuscular (IM) and subcutaneous (SC) routes (Figure 1). In the majority of cases, this allows the formation of a local depot at the injection site, from where the antigen and adjuvants (if present) can be drained to the local LN. This process can happen passively or through immune cell capture and transport to the LN, depending on the characteristics of the vaccine formulation. Transdermal (TD) and SC injections have been presented as alternatives to IM immunisation precisely due to the advantages they may present in terms of vaccine drainage to the lymphatics and overall immunogenicity [43]. However, the presence of adjuvants in vaccine formulations has occasionally led to increased levels of local side effects observed following SC and TD administration, maintaining the preference for IM injections over the other parenteral alternatives (Figure 1) [44].

Despite their widespread use, parenteral routes of immunisation present significant limitations, namely, in terms of the financial and human resources required for preparing, administering, and disposing of injectable materials, and the risks associated with needle-stick injuries and sharp waste management. For these reasons, mucosal routes of administration have also been explored for vaccines, particularly considering the fundamental role played by the mucosal-associated lymphatic tissue (MALT) in eliciting mucosal immunity at a local level [45]. The oral and nasal routes have been most widely studied in this regard (Figure 1), with a focus on the development of local immune response following antigen presentation by APCs such as macrophages and dendritic cells (DCs) to tissue-resident T and B cells. This type of immune response is particularly important, as evidenced through the secretion of antigen-specific immunoglobulin A (IgA) antibodies which are capable of recognising the antigen at the entry site, and which therefore prevent the further spread of the pathogen in the body [46,47]. This level of protection is often difficult to achieve with parenteral immunisation strategies, so it is particularly important for the scientific community to focus efforts on the development of formulations for mucosal vaccine administration.

Regarding the oral route, vaccine developers must consider the harsh gastrointestinal (GI) environment when designing a formulation (Figure 1). Antigen protection against low gastric pH, high enzymatic presence and significant mucus layer throughout the tract is essential, particularly when developing modern vaccines with peptide, protein or nucleic acid antigens [48]. Oral vaccination has proved to be a successful approach when direct delivery to the site of infection in the GI tract may have greater impact on eliciting immune responses where required. For instance, the success of the oral polio vaccine (OPV) in reducing infection and the transmission of polio has been attributed to local immune responses in the intestinal mucosa, where poliovirus replicates [49].

An alternative to the GI obstacles would be to focus on the intranasal (IN) route of administration, which also presents great advantages in terms of vaccine delivery, including high vascularisation of the nasal mucosa and rapid absorption of antigens to the systemic circulation (Figure 1) [50]. Also, many pathogens enter through this route to induce life-threatening respiratory diseases, making this region even more important and attractive in terms of developing vaccines against these infectious agents. For instance, the IN delivery of a coronavirus vaccine may provide stronger mucosal immunity in the nose and lungs, offering protection at the site of entry [51]. Indeed, a chimpanzee adenovirus vectored SARS-CoV-2 vaccine elicited strong humoral and cellular responses in the nasal mucosa in a mouse model [52]. Several clinical trials for IN vaccines are now underway, which will reveal if these responses also translate to humans. Practical advantages of a nasal spray vaccine as a less invasive method of administration have also been shown previously with the influenza FluMist vaccine. Nevertheless, the limitations of the IN administration route, including the rapid mucus clearance leading to a short residence time of the antigens in the nasal mucosa and the size-restricted permeation of antigens and adjuvants across the epithelial barrier, should also be considered when developing vaccine formulations [50]. There is also a pressing need for new adjuvants that can be safely administered through mucosal routes (as opposed to alum, which cannot be used in these approaches) to improve the immune response generated against recombinant subunit antigens.

It is worth mentioning the recent interest in the transdermal (TD) route for vaccination purposes (Figure 1). The skin is the largest human organ, with an extensive immune cell population and close access to the bloodstream and lymphatic system, evidencing an enormous potential for targeted vaccine delivery. Given the particular structure and composition of the skin, with its external stratum corneum being practically impermeable to drugs and antigens, the main challenges in developing vaccine formulations for the TD route are related to overcoming this penetration issue. Over the years, researchers have taken various approaches to tackle this challenge, mostly through the use of penetration enhancers or physical processes to disrupt this barrier and allow drug and antigen delivery. Microneedle (MN) arrays, i.e., patches containing a variable number of needle-like projections in various shapes and dimensions (generally below 1 mm in height), have gained attention in the last few decades, also for immunisation purposes (Figure 1) [32,53]. These structures provide a painless alternative to vaccine TD delivery, allowing the interaction of antigens and adjuvants with the dermal immune cell population, and facilitating their access to draining lymphatics, potentially generating local and systemic immune responses through this route.

## 4. Delivery Systems of Vaccines

The appropriate design of a delivery system for vaccines is as crucial as the choice of a pertinent administration route for enhancing immune responses. A well-designed delivery system can significantly improve the bioavailability of viral antigens by ameliorating cellular uptake, conferring metabolic stability and targeting relevant tissues. In this section, various vaccine delivery systems will be discussed, highlighting the pros and cons of each system, with a focus on the recently introduced nanocarriers and new delivery technologies.

### 4.1. Viral Vectors

**Figure 2:**
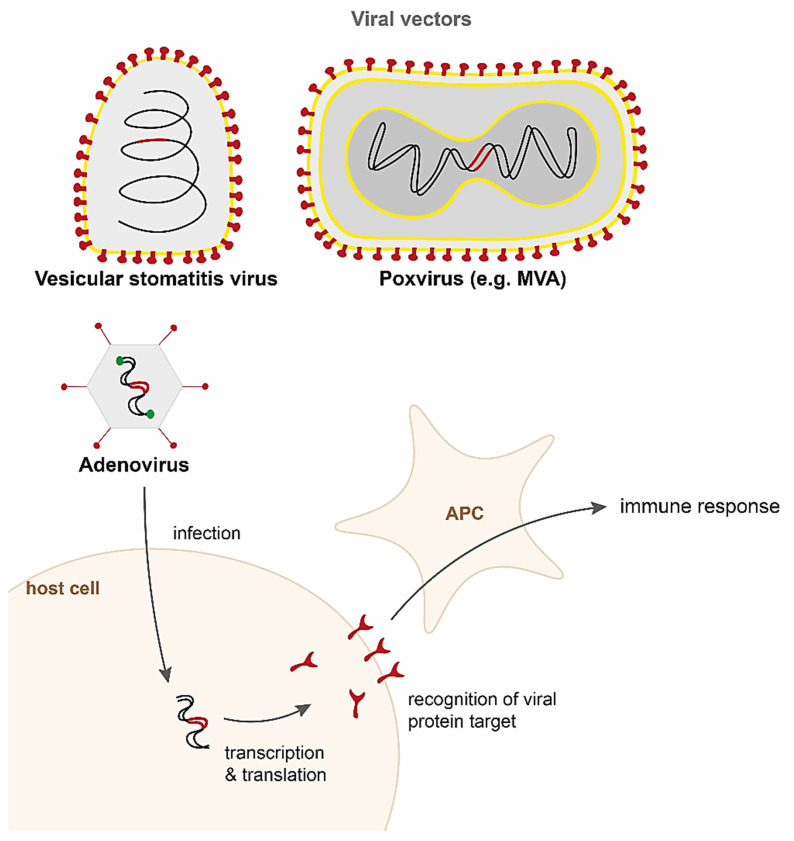
Delivery of vaccines by viral vectors. Schematic diagrams representing the viral vectors vesicular stomatitis virus (VSV), poxviruses, and adenoviruses encode the target viral antigen (red). Entry into the host cell is driven by proteins expressed by the vector. The target viral antigen is expressed and recognised by the host immune system. Antigen processing cells (APC) engulf the antigens and activate the adaptive immune response to elicit antibody and T cell responses.

Although the idea of using viral vectors for delivering vaccines is not a recent one, the first recombinant viral vector vaccine, developed against Ebola virus, was only approved for medical use in Europe and the US in 2019 [54,55]. The first demonstration of a viral vectored vaccine in the early 1980s was a recombinant vaccinia virus (VACV) expressing the hepatitis B surface antigen (HBsAg), which was shown to induce protective immune responses against hepatitis B virus in a nonhuman primate model [56,57]. The technology relies on viral vectors encoding for pathogen antigens being delivered to the host, after which the antigens are expressed and an immune response is mounted against the target pathogen (Figure 2). Viral vectors can be either replication-competent or nonreplicating, although the latter generally elicit weaker immune responses. The greatest advantage of viral vectors is their high immunogenicity; however, concerns about the safety of replication competent vectors has hindered their rapid development. Recently, newer generation single-cycle vectors that amplify antigen genes without the risk of infection are being investigated [58].

Numerous viruses are currently undergoing clinical and preclinical trials as vectors for vaccines including adenoviruses, poxviruses (e.g., Modified vaccinia Ankara, MVA; horsepox virus), lentiviruses (e.g., human immunodeficiency virus, HIV), rhabdoviruses (e.g., vesicular stomatitis virus, VSV; rabies virus), paramyxoviruses (e.g., measles virus, Newcastle disease virus, Sendai virus), flaviviruses (e.g., Yellow Fever virus), and herpesviruses (e.g., cytomegalovirus, CMV). There are currently six viral vector vaccines licensed globally, including four against COVID-19 (adenovirus vectors in Oxford-AstraZenica, Sputnik V, Janssen, and Convidecia) and two against Ebola (vesicular stomatitis virus vector in rVSV-ZEBOV, and adenovirus and MVA in Zabdeno/Mvabea). A comprehensive review of the whole range of vectors exceeds the scope of this review and have been discussed in detail previously [59,60,61,62,63,64,65]. Here, we will focus on three of the most common vectors that are currently being developed against COVID-19—adenovirus, VSV, and MVA (Figure 2).

#### 4.1.1. Adenoviruses

Adenoviruses are one of the most common vectors used and in trials for vaccine delivery. Belonging to the Adenoviridae family of viruses, they are nonenveloped double-stranded DNA viruses with genomes of approximately 30–40 kb in length (Figure 2). Adenoviruses are widespread across the animal kingdom, and currently there are over 80 human adenoviruses (HAdVs) types. They are categorised into seven species, A to G, with species C serotype 5 (Ad5) being most highly prevalent [66]. The tropism of the virus is determined by the targeted cell host receptor, and the numerous types allow for a broad tropism. For instance, species C HAd5 and HAd2 bind to the coxsackie adenovirus receptor (CAR), expressed on endothelial and epithelial cells [67]; B species HAd35 binds to CD46, ubiquitous to many cells [68]; and B1 species HAd3 binds to CD80/CD86 expressed on APCs [69]. In addition, tropism can be altered by modification of the capsid to create chimeric Ad viruses [70]. The vector can be replication-competent or replication-defective, the latter typically by the removal of early transcript 1A (E1A) and E1B, both which are required for replication. In addition, E3 is often deleted as it is not required for replication in cell culture, and deletion of E4 prevents leaky expression of the early genes [71]. HAdVs are produced at high titres in mammalian cell culture, with E1 proteins provided in trans [72]. Although the vector has a relatively small insert size of 7.5 kb, minimal adenovirus ‘gutless’ vectors, with most viral genes removed, have also been developed to allow insertions of foreign sequences of up to 38 kb [73]. The viral genome is episomal, but there is some risk of integration as viral replication and transcription occurs in the nucleus of the host cell.

Much work in mouse models has shown that HAdVs elicit potent antibody and T cell immune responses [70]; however, the serotype contributes to slight variations in the phenotype and functional properties of memory T cells elicited by the vector. Innate immune responses including production of pro-inflammatory cytokines and activation of complement has also been reported [74]. However, the ubiquitous nature of Ad5 in humans leads to the attenuation of immune responses due to pre-existing immunity, demonstrated in Ad5 vectored HIV vaccine trials [75,76]. A single dose of an Ad5 vectored vaccine against COVID-19 showed a dose-dependent production of neutralising antibodies, and specific T cell responses. However, in keeping with previous observations, in individuals with a high concentration of Ad5-specific antibodies, T cell responses were attenuated, particularly at lower doses of the vaccine [77].

Therefore, adenovirus types that are rarer such as HAd26 and HAd35 have been developed as vectors to combat this. A preclinical study in mice showed that the HAd26 vectored COVID-19 vaccine induced strong antibody and T cell responses [78]. The replication-deficient Ad26.COV2.S (Janssen, Beerse, Belgium) vaccine has similarly shown robust protection against symptomatic COVID-19 in human trials, with potent neutralising antibodies and induction of T cell responses against multiple SARS-CoV-2 variants of concern [79,80,81]. The immune response elicited seems to depend on the vector type used for the delivery of the vaccine. For instance, a Zika virus vaccine expressing the Zika proteins precursor membrane (prM) and envelope (E) via HAd4 or HAd5 vectors (Ad4-prM-E or Ad5-prM-E) both showed protection against disease in a mouse model. However, the Ad5-prM-E vaccine induced both humoral and T cell immunity, while the Ad4-prM-E elicited only T cell responses [82]. Indeed, administration of the Ad5-prM-E alongside a UV-inactivated HAd4 vector reduced the anti-Zika antibodies, suggesting that the HAd4 capsid could skew the immune profile towards T cell responses [83].

Nonhuman adenoviruses such as bovine adenoviruses (BAdV) and chimpanzee adenoviruses (ChAd) provide an alternative avenue to bypass pre-existing immunity; indeed, the ChAd hypervariable regions of the immunogenic capsid hexon protein were shown to be sufficiently different from HAd5 [84], and pre-existing HAdV antibodies did not cross-react with BAdV-3 vector [85]. BAdV vectors targeting influenza proteins elicited strong humoral and cell-mediated responses in preclinical small animal models [86,87]. Among the nonhuman adenoviruses, ChAd vectors have progressed the furthest in terms of use in humans. An Ebola vaccine delivered via a ChAd3 vector (ChAd3-EZO-Z) showed robust antibody and CD8 T cell responses in two small human trials, with no adverse effects [88,89]. The ChAdOx1 vector, derived from ChAdY25, has been successfully used in the Oxford-AstraZeneca COVID-19 vaccine, with 62% efficacy after two doses [90]. Both antispike neutralising antibodies and immune cell activation against SARS-CoV-2 were measured [91], and a study reported the activation of a diverse T cell receptor (TCR) repertoire against different areas of the spike protein suggesting a robust T cell response [92]. A better understanding of vector-induced immunity in simian and alternative HAd vectors is required for the development of new viral vaccines and to evaluate their use in repeated booster doses.

#### 4.1.2. Poxviruses

Poxviruses are large, enveloped double-stranded DNA viruses, with genomes of approximately 190 kb, and a high capacity of 25 kb for transgene insertion (Figure 2). The most famous, vaccinia virus, used as live vaccine against smallpox, is highly effective at preventing disease. However, vaccinia was also associated with a range of adverse reactions, more so than most other vaccines [93]. The Modified vaccinia Ankara (MVA), a highly attenuated strain with approximately 15% of the genome deleted, has been investigated as a safer and effective vector against many viral diseases [94,95]. MVA is a nonreplicating vector that can be produced at high titres, and although it is generally well tolerated, high doses of vector caused some adverse effects [96].

MVA has been shown to induce potent humoral responses [97], and robust CD8 T cell responses comparable to other vaccinia strains [98]. As many viral genes that usually allow for host immune evasion are deleted in MVA, the virus shows enhanced antigen presentation and immunogenicity, leading to an increase in proinflammatory cytokines and improved migration of monocytes and leukocytes [99,100]. Removal of immunomodulatory genes in MVA can further improve immunogenicity. For instance, deletion of the IL-18 binding protein gene C12L, increased CD8 and CD4 T cell responses to vaccinia epitopes by up to three-fold, with greater protection against vaccinia infection in a mouse model [101]. Recently, the repair of two mutated or missing host range genes (C16L/B22R and C12L) were shown to restore replication of MVA in human cell lines, suggesting that MVA vaccines can also be engineered into replicating vectors [102], which could further improve immune responses.

MVA vectors are being developed for influenza and other respiratory viruses, and protection against these viral infections has been demonstrated in preclinical animal models [94]. Additionally, a Phase I/II trial of MVA vectored vaccine targeting influenza HA (rMVA-HA) showed induction of neutralising antibodies and HA-specific T cell responses [103]. Another recombinant MVA vector targeting influenza nucleoprotein and matrix protein 1 (MVA-NP + M1) in individuals over 65 was deemed to be well-tolerated, although the trial was not sufficiently large to determine its efficacy [104,105]. An MVA vectored SARS-CoV spike vaccine elicited neutralising antibody responses in mouse, rabbit, and macaque models [106,107]. Similarly, high humoral responses were observed in mice administered an MVA vectored MERS-CoV spike vaccine [108], and complete seroconversion and MERS-CoV spike-specific T cell responses measured in at least 83% of individuals given the same vaccine in a small Phase 1 clinical trial [109]. Although no MVA vaccines against COVID-19 have yet entered human trials, several studies have shown to induce strong and specific cellular responses against SARS-CoV-2 spike in mice [110,111,112]. An MVA vectored vaccine expressing prefusion stabilised SARS-CoV-2 spike induced robust antibody and CD8 T cell responses and offered protection from lung infection in a macaque model [113]. The development of MVA vectored vaccines soon after the emergence of SARS-CoV, MERS, and SARS-CoV-2 suggests that this platform can be used for rapid response against emerging viruses.

#### 4.1.3. Vesicular Stomatitis Virus

Vesicular stomatitis virus (VSV) is an enveloped single-stranded negative sense RNA virus belonging to the *Rhabdoviridae* family (Figure 2). The development of a reverse genetics system in 1995 allowed for the virus to be grown to high titres as well as engineer recombinant VSV (rVSV) to express foreign sequences [114]. The genome size is approximately 11 kb, with a relatively small insert size of 5 kb. It is typically used as an attenuated vector, achieved by deletion of the viral glycoprotein G, mutating the viral matrix protein M, or rearranging the order of viral proteins or insertion of exogenous proteins [115]. The glycoprotein G determines the tropism of the virus, which can be altered by replacing with a transgene.

VSV infects livestock, but rarely humans. This implies a low risk of pre-existing immunity; however, antivector immunity was detected in one-third of individuals given the vector, which may cause issues in situations where multiple doses or multiple VSV vaccines are administered [116]. Interestingly, replacing the G protein with a glycoprotein of lymphocytic choriomeningitis (VSV-GP) in a vector expressing ovalbumin (OVA) showed lower neutralising antibody titres compared to a standard VSV-G-OVA vector in mice, with no loss of efficacy upon booster doses [117]. This suggests that altering the vector can help overcome vector-specific immunity. In addition, there have been some concerns of safety due to risk of neurovirulence observed in animal models. For instance, mice infected intranasally with wild-type VSV showed CNS infection via infection of the olfactory neurons [118,119]. However, no neurovirulence was observed in macaques infected intranasally with rVSV, suggesting that no vector-associated pathogenesis occurs in nonhuman primate models [120].

One of the earliest preclinical studies in the 1990s showed that a rVSV vectored influenza vaccine elicited high levels of neutralising antibodies in mice [121]. The first human clinical trial was undertaken nearly two decades later with a rVSV vectored HIV vaccine, in which all vaccinated individuals developed HIV-specific antibodies after two doses, and HIV gag protein-specific T cell responses were detected in more than half of the individuals in the highest dose group [122]. There is currently one licensed rVSV vectored vaccine against Ebola (rVSV-ZEBOV). In a Phase 3 clinical trial in Guinea during the Ebola outbreak in 2014–15, rVSV-ZEBOV showed good efficacy by employing the ring vaccination strategy [123]. The vaccine induced robust humoral responses, while the highest dose also elicited modest T cell responses [124]. An rVSV vectored MERS-CoV spike vaccine showed rapid and potent neutralising antibody responses in a macaque model, although antibody titres declined by six weeks postvaccination [125]. An rVSV vaccine expressing SARS-CoV-2 spike protected against SARS-CoV-2 challenge in a hamster model and reduced viral titre in the lungs and upper respiratory tract [126]. Similarly, a replication competent VSV-SARS-CoV-2 vaccine expressing modified spike protein also showed protection against lung infection in mice, with a high titre of neutralising antibodies. Indeed, these serum antibodies were protective against disease in nonvaccinated mice [127]. VSV vectors have generally been shown to induce strong neutralising antibody responses, but lower CD8 and CD4 T cell immunity [59]; however, whether this is sufficient for protective immunity still needs to be determined.

### 4.2. Nonviral Vectors

**Figure 3:**
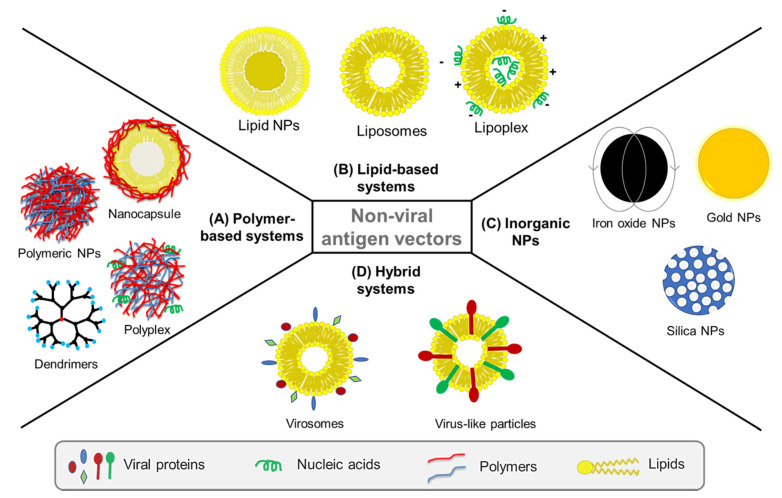
Nonviral vectors used for delivery of vaccines against viruses are classified into: (**A**) polymer-based systems such as polymeric nanoparticles (NPs), polyplexes, polymeric dendrimers and polymeric nanocapsules, (**B**) lipid-based systems such as liposomes, lipid NPs and lipoplexes, and (**C**) inorganic NPs such as iron oxide, gold, and mesoporous silica NPs. In addition to nonviral vectors, (**D**) hybrid systems such as virosomes and virus-like particles (VLPs) have been also developed to combine nonviral systems like liposomes with viral elements, for instance decorating liposomes with viral glycoproteins to imbue the system with viral immunogenicity.

As the main aim of a vaccine is to be immunogenic, preferably at low dose and dosing frequency, it is important for a vaccine delivery system to present the viral antigen in an effective and sustained manner to trigger the desired immune response. In essence, nonviral vectors offer a great platform for the development of such effective vaccine delivery systems. Safety and efficacy, protection of antigen from degradation, and potential to act as adjuvants are some of the advantages nonviral vectors present for vaccine delivery [128]. In the last few decades, nanocarriers have been explored as nonviral vectors and as alternatives to conventional vaccines against infectious diseases [129,130,131,132,133,134]. For instance, polymeric and inorganic nanoparticles, dendrimers, liposomes and most recently virosomes, have been used for sustained delivery of viral antigens and adjuvants, protecting viral proteins against degradation, targeting host cells, and promoting the stimulation of immune cells (Figure 3).

Beyond their ability as vaccine delivery vehicles, the nanoscale size and ability to target APCs and stimulate different immune cells depending on the biomaterials used in their composition make nonviral vectors suitable as adjuvants, antigenicity enhancers and immunity boosters [135]. The biological properties of nanocarriers, and thus, their interaction with immune cells, is influenced by their physicochemical characteristics including particle size, shape, surface chemistry, hydrophobicity/hydrophilicity and steric effects of particle coating [136]. Engineering nanocarriers with respect to these properties is therefore crucial for their role as vaccine delivery vehicles and as potential vaccine adjuvants [137]. Various types of nonviral nanocarriers including polymeric, lipid-based and inorganic ones have been studied in this regard (Figure 3).

Other advanced delivery systems based on supramolecular hydrogels and microneedles have also been recently introduced as depot formulations for sustained and localised delivery, to enhance and prolong immune responses to vaccines, which are discussed under Section 4.4. Other Advanced Vaccine Delivery Systems.

#### 4.2.1. Polymer-Based Systems

Both natural and synthetic polymers have been widely used as drug delivery systems thanks to their physicochemical tunability, versatility of molecular design, biocompatibility and biodegradability, making them promising vehicles for the controlled and targeted delivery of antigens [138]. Antigens can either be encapsulated or adsorbed on the surface of polymers. The encapsulation of an antigen protects it from exposure to metabolic enzymes as well as the harsh GI environment, if the oral route is chosen for administration, whereas antigen adsorption avoids exposure to harmful organic solvents or extreme pH during the formulation process [139]. Over the years, polymeric systems such as nanoparticles, polyplexes, dendrimers and nanocapsules have been developed for delivery of vaccines against viruses.

##### Polymeric Nanoparticles (NPs)

Polymeric NPs have gained great attention for their applications as vaccine delivery vehicles due to their biocompatibility, biodegradability and ease of preparation [140]. According to the materials used in their composition, polymeric NPs can be divided into natural polymer NPs and synthetic polymer NPs [130,132]. Both types of NPs have been studied over the years as nonviral antigen carriers to deliver a wide range of antigens including hepatitis B virus (HBV) antigen [141,142], influenza virus [143], HIV, hepatitis C, dengue virus [131], and Ebola virus [144].

Chitosan, dextran, hyaluronic acid and beta-glucans are among the most commonly used natural polymers in the development of vaccine delivery systems [145]. These biomaterials are particularly attractive since many of them appear naturally in the structure of some microorganisms, making them easily recognisable by immune cells and therefore increasing the possibility of generating an immune response against the loaded antigen [146,147]. Chitosan is a naturally occurring cationic biopolymer which interacts with the negatively charged cellular membrane of the epithelium [148]. The adsorption of chitosan NPs with the nasal and intestinal mucosa is enhanced, significantly inducing immune responses against these nanocarriers. For example, Prego et al. developed chitosan NPs for the delivery of recombinant HBV antigen [141]. Researchers intramuscularly injected the NPs in mice and observed a 9-fold higher amount of HBV-specific Immunoglobulin G (IgG) antibodies than with the conventional aluminium-adsorbed vaccine. More recently, Cordeiro et al. developed carboxymethyl-β-glucan (CMβG)-chitosan nanoparticles for delivery of OVA as a model antigen [145]. In this study, a single vaccine dose subcutaneously injected in mice induced T cell proliferation and antibody responses comparable to those achieved with alum-adsorbed ovalbumin. Dacoba et al. reported the preparation of NPs by covalently bonding an HIV antigen, tethered via the protease cleavage site peptide PCS5, to chitosan or hyaluronic acid and further associating it with oppositely charged polymers such as dextran sulphate or chitosan and polyinosinic:polycytidylic acid (poly(I:C)) [149]. The results showed that all NPs systems elicited high anti-PCS5 antibodies and NPs containing PCS5 conjugated and poly(I:C) induced the strongest activation of antigen-presenting cells. El-Sissi et al. developed chitosan NPs loaded with Rift Valley Fever Virus (RVFV) inactivated antigen and studied the effect of this formulation in the vaccination of Swiss albino mice [150]. The results indicated that antigen-loaded chitosan NPs produced enhanced phagocytic activity of peritoneal macrophages and neutralised antibody levels against RVFV and IgG levels against RVFV nucleoprotein, in comparison with adjuvant-free RVFV inactivated antigen. These are a few examples among many in which NPs based on natural polymers have been used for the delivery of viral antigens, which have been extensively reviewed by other researchers [151,152,153,154].

NPs have also been developed for the delivery of antigenic viral components using synthetic polymers, the most investigated of which include poly (lactic-*co*-glycolic acid) (PLGA), poly(glycolic acid) (PGA), poly(lactide-*co*-glycolide) (PLG), poly(lactic acid) (PLA), poly(alkyl cyanoacrylate) (PACA) and polyanhydrides. The properties of polymers vary depending on their composition. For example, PLA produces dense, flexible structure that degrades slowly, while PGA is stiff but degrades rapidly. On the other hand, PLGA has properties in-between those shown by PLA and PGA. Thus, altering the composition or ratio of copolymers used during the NP synthesis process, can enable vaccine release and uptake control [132,155]. For instance, Thomas and coworkers studied mixed systems of synthetic polymers of PLA and PLGA, with various ratios of the two, as a delivery system for HBV surface antigen (HBsAg) through the pulmonary route [142]. The results showed that a higher presence of PLA produced NPs with larger size, which were taken up increasingly by rat alveolar macrophages, leading to an increase in cytokine secretion. In another study, Ross et al. showed that a recombinant H5 hemagglutinin trimer (H53) encapsulated into polyanhydride NPs induced high neutralising antibody titres and enhanced CD4^+^ T cell recall response in mice, inducing protective immunity against H5N1 influenza [143]. Rietscher and coworkers evaluated the potential of a vaccine delivery system made of hydrophilic polymer polyethylene glycol (PEG)-poly(allyl glycidyl ether) (PAGE)-*b*-PLGA (PPP) loaded with model antigen OVA [156]. In their in vitro studies, researchers observed a significant enhancement in T cell activation by APCs when antigen was delivered via PPP NPs in comparison with PLGA NPs or free OVA. Further, results showed that the subcutaneous application of PPP-OVA-NPs even without adjuvants induced potent CD8 T cell-mediated OVA-specific cytotoxicity in vivo, as compared to that caused by PLGA-OVA-NPs or OVA alone. Knight et al. demonstrated that a pH-responsive NPs vaccine loaded with OVA and CpG DNA adjuvant increased the magnitude and longevity of pulmonary CD8^+^ tissue-resident memory T cell response in mice [157]. The pH-responsiveness was given by a diblock copolymer made of hydrophilic copolymer of dimethylaminoethyl methacrylate (DMAEMA) and pyridyl disulphide ethyl methacrylate (PDSMA), and hydrophobic copolymer of propylacrylic acid (PAA), butyl methacrylate (BMA) and DMAEMA. Antigen-loaded NPs enhanced the activation of pulmonary APCs and assisted antigen persistence in the lungs. A single IN dose of the NPs vaccine provided protection against respiratory virus in both sublethal (vaccinia) and lethal (influenza) infection models for up to 9 weeks after immunisation.

##### Polyplexes

Polyplexes are complexes formed by polymers and nucleic acids [158]. Neutral, cationic and amphiphilic polymers have been used to produce polyplexes for gene delivery applications (Figure 3A) [159]. Cationic polymers provide better delivery systems due to their easy electrostatic interactions with negatively charged oligonucleotides and cellular membranes. Synthetic cationic polymers such as PLA, poly-L-lysine (PLL), polyetherimide (PEI), poly(amidoamine) (PAMAM), poly(2-dimethylaminoethylmethacrylate) (PDMAEMA), as well as carbohydrate-based natural polymers such as chitosan, have been used to synthesise polyplexes. Moreover, strategies such as PEGylation and functionalisation with targeting ligands are employed to improve their transfection efficiency and circulation times [160].

In one study, Demoulins and coworkers used a polyplex made of PEI or histidylated PEI and a self-amplifying mRNA encoding influenza virus hemagglutinin and nucleocapsid [161]. The polyplex system successfully delivered the mRNA to DCs eliciting both humoral and cellular immune responses and improved the efficacy of mRNA vaccine. However, toxicity remains a challenge with the high molecular weight of PEI and thus alternative systems have been researched [162]. Li et al. studied the ability of two types of cyclodextrin (CD)-PEI polymers, prepared using different ratios of cyclodextrin to PEI, as intranasal mRNA vaccine carriers [163]. The conjugate CD-PEI nanocomplex delivery system was shown to traffic to lymph nodes with higher efficiency and to stimulate potent humoral and cellular immune responses. Polyplexes of polymers such as PDMAEMA have also been demonstrated to improve transfection efficiency of mRNA vaccines [164].

Recently, polyplexes formulations were also explored for challenging viral infections such as HIV-1 infection. Moyo and coworkers used the cationic polymer polyethyleneimine (PEI)-based self-amplifying mRNA vaccine encoding HIV-1 proteins to enhance cellular uptake of mRNA and induce potent T cell responses in BALB/c mice [165]. A single injection induced polyfunctional CD4^+^ and CD8^+^ T cell responses that lasted for at least 4 months postadministration and controlled HIV-1.

##### Polymeric Dendrimers

Dendrimers are highly branched, three-dimensional, star-shaped polymeric macromolecular structures (Figure 3A). These are synthesised from a polyfunctional core, e.g., ammonia or ethylenediamine, which dictates the three-dimensional shape of the molecule. Repeat units, such as PAMAM, polyamino acids, polyphenyls, polyporphyrins and polyethers, are added to the core and react with its functional groups. Each layer of the repeat units thus produces branching and increases the number of surface functional units. In the final step, the dendrimer is capped with a layer that provides the desired surface chemical properties of the system. The interior layers are suitable for encapsulation of therapeutic or biomolecule while the exterior layer is made of functional groups which are useful for conjugation of these biomolecules and targeting moieties. Thus, by altering the nature of the core and repeating units, the number of layers, and the composition of the surface layer, it is possible to synthesise dendrimers with desired chemical and biological properties. Due to these unique characteristics, this class of polymeric nanomaterial has found applications in drug, gene and vaccine delivery [166].

Dendrimers exhibit efficient immune-stimulating properties, and thus can act as adjuvants and can enhance the efficiency of vaccines [167,168]. In a study, Asgary et al. synthesised a nonlinear globular G2 dendrimer comprising citric acid and polyethylene glycol 600 (PEG-600) and evaluated the adjuvanticity of NPs containing the rabies virus inactivated antigen in a mice model [169]. They observed that dendrimer-based formulations enhanced immune responses, induced high neutralising antibodies against rabies virus, and led to higher survival rate of mice. Chahal and coworkers found that a single dose of dendrimer encapsulated with multiple antigens was able to produce strong antibody and T-cell responses against Ebola virus, H1N1 influenza, and *Toxoplasma gondii* [170].

##### Polymeric Nanocapsules (NCs)

Nanocapsules (NCs) are composed of an inner lipid core usually stabilised by phospholipids and an external polymeric shell (Figure 3A). The main advantage of NCs is the opportunity to load hydrophobic adjuvant molecules in the core while antigens are displayed on the surface, associated with the external polymers through different types of interactions [171]. NCs coated with different polymers such as chitosan, inulin, protamine, polyarginine and beta-glucans, have been explored by Alonso and coworkers, with promising results [172,173,174]. In these studies, the authors demonstrated the potential to engineer these NCs for efficient lymphatic targeting, particularly through optimisation of particle size, surface charge and selection of different coating polymers. The results showed that small NCs (below 100 nm) with neutral or positive surface were able to drain efficiently to the closest lymph node following subcutaneous injection to the mice footpad. Additionally, protamine-coated NCs were able to efficiently deliver recombinant hepatitis B antigen to immune cells in mice, leading to a protective humoral response.

#### 4.2.2. Lipid-Based Systems

A variety of lipid-based systems have been developed as antigen carriers, with particular focus on emulsions of micro- and nano- metric size. In fact, the first adjuvant approved for human vaccines after alum was MF59^®^, an emulsion of squalene oil, Tween^®^ 80 and Span^®^ 85 included in Fluad^®^, a flu vaccine developed by Novartis [175]. Further research led to the development of other adjuvant emulsions such as AS04, approved for a human papilloma virus (HPV) vaccine, AS03, approved for use in Pandemrix^®^ during the 2009 H1N1 influenza pandemic until 2015 [176], as well as AS01 and AS02, used in a malaria vaccine that reached clinical development and recommended by WHO for children [177,178,179]. Owing to their excellent surface activity, biocompatibility and biodegradability characteristics, amphiphilic lipids were widely used to develop lipid-based systems such as liposomes, lipid nanoparticles and lipoplexes, which attracted researchers for their application in biomedicine including in vaccine delivery.

##### Liposomes and Lipoplexes

Liposomes were the first lipid-based nanocarrier platform to be developed for drug delivery, and one of the most explored vehicles in drug and antigen delivery [180,181,182,183]. Liposomes are self-assembled nanostructures, consisting of unilamellar or multilamellar vesicles composed of amphiphilic lipids and water (Figure 3B) [184,185]. Like polymeric NPs, liposomes are also biocompatible and biodegradable. Moreover, they can incorporate hydrophobic agents within their lamellae and hydrophilic agents in their aqueous core, thanks to their amphiphilic nature. These features provide advantages for these systems as delivery vehicles for drugs, antigenic proteins and peptides. Additionally, particle size and surface charge of the liposome bilayer can be tuned and functionalised with ligands for targeted delivery applications [186,187]. Based on their surface charge, liposomes are divided into cationic, anionic, and neutral. Cationic liposomes are much more efficient than the other types, especially for sustained antigen release, since the positive charge enhances the interaction with the negatively charged cellular membranes [188].

There are several liposomal products that have gained marketing authorisation globally for the treatment of various diseases, including infections and cancer. In addition to delivering antigen, liposomes can act as adjuvants. Recently, a liposomal formulation containing monophosphoryl lipid A (MPLA) and the saponin QS-21 was approved as an adjuvant for a recombinant zoster vaccine [189]. Tokatlian et al. developed a delivery system consisting of synthetic liposomes with a gp140 trimer, BG505 MD39, covalently coupled on its surface, to study the effect of trimer density and vesicle stability on vaccine-induced humoral responses in mice [190]. They observed that immunisation with covalent MD39-coupled liposomes, as compared to those with soluble MD39 trimers, led to increased antigen-specific T follicular helper cell responses and significantly higher MD39-specific IgG responses. Two vaccines for the prevention of herpes zoster are currently available, namely, Zostavax (ZVL) and Shingrix (herpes zoster subunit vaccine (HZ/su)). Herpes zoster, also known as shingles, is caused by the reactivation of the varicella-zoster virus (VZV), the same virus that causes varicella (chickenpox). Zostavax (ZVL) is a live, attenuated vaccine, whereas Shingrix^®^ (herpes zoster subunit vaccine (HZ/su) is an adjuvanted recombinant subunit vaccine [191,192]. ZVL was approved by the Food and Drug Administration (FDA) in May 2006 while HZ/su was approved in October 2017 for the prevention of herpes zoster in individuals 50 years of age and older. Shingrix^®^ is superior to Zostavax in both safety and efficacy, and is based on a liposome delivery system consisting of 1,2-dioleoyl-*sn*-glycero-3-phosphocholine (DOPC)/cholesterol/monophosphoryl Lipid A (MPLA) alongside saponin Quillaja saponaria Molina fraction 21 (QS-21) as an adjuvant and varicella zoster virus (VZV) glycoprotein E (gE) as the antigen. Immunogenicity, efficacy, and safety data indicated HZ/su significantly reduced the risk of developing herpes zoster by more than 90% and thus use of the vaccine is recommended to all immunocompetent patients older than 50 years to prevent herpes zoster. In addition, as a subunit vaccine, it also showed good safety and efficacy in people with immunocompromising diseases, including HIV carriers [193].

Lipoplexes are also lipid-based carrier systems, which involve complexes formed by lipids and nucleic acids (Figure 3B). Cationic lipids, such as 1,2-di-*O*-octadecenyl-3-trimethylammonium-propane (DOTMA) and 1,2-dioleoyl-3-trimethylammonium-propane (DOTAP), and zwitterionic lipids, such as 1,2-dioleoyl-*sn*-glycero-3-phosphoethanolamine (DOPE), have been used for mRNA vaccine delivery. Previous studies demonstrate that the physicochemical characteristics and biological activity of lipoplexes can be tuned by changing the lipid components, ratio of cationic lipid to mRNA, and ionic conditions [194,195]. Hattori and coworkers evaluated the efficiency of a lipoplex system consisting of mannosylated liposome/model antigen OVA-encoding pDNA (pCMV-OVA) for gene delivery to DCs [196]. Using in vitro study, they showed that the lipoplex could transfer pCMV-OVA more efficiently than cationic liposomes. Further in vivo study by the authors indicated that the mannosylated lipoplex systems provided enhanced OVA-specific cytotoxic T lymphocyte (CTL) activity than the conventional lipoplex or naked pCMV-OVA. Rhee et al. identified a B cell epitope peptide, from the HA protein of the H5N1 A/Vietnam/1203/2004 strain, which can potently induce production of epitope-specific antibodies. They reported that the immunization with a complex of B cell epitope of HA protein and Lipoplex(O), which is MB-ODN 4531(O), a natural phosphodiester bond CpG-DNA co-encapsulated in a phosphatidyl-*b*-oleoyl-*c*-palmitoyl ethanolamine (DOPE):cholesterol hemisuccinate (CHEMS) complex (1:1 ratio), completely protected the mice from the challenge by a lethal dose of recombinant H5N1 virus (rH5N1 virus) [197]. Lipoplexes are still at early stages of research and although promising, more work is required to understand the effect of lipid components and charge on the cellular delivery of nucleic acid-based antigens, and impact of this on immunisation effectiveness. 

##### Lipid Nanoparticles (LNPs)

LNPs are generally composed of different types of lipids with different functions. Cationic lipids are usually added for mRNA complexation, while ionisable lipids can facilitate in vivo delivery and endosomal escape. Other components such as phospholipids, cholesterol and PEGylated lipids can also be added to contribute to improve NP properties such as stability, tolerability and biodistribution (Figure 3B) [198,199,200].

Therefore, LNPs have gained interest for delivery of modern vaccines in recent years, particularly considering their potential for improved intracellular delivery [201]. Moderna’s mRNA-1273 vaccine and Pfizer-BioNTech BNT162b2 vaccine, which have received Emergency Use Authorisation (EUA) by the MHRA in the UK, the EMA in the EU and the FDA in the US for use in adults to prevent coronavirus disease caused by SARS-CoV-2, are based on this type of NP [202]. In these products, LNPs are composed of an ionisable lipid for mRNA complexation and NPs assembly, a PEGylated lipid to increase NPs circulation time, cholesterol for increased stability and other phospholipids for structural support. In terms of the antigen, in both vaccines the LNPs encapsulate nucleoside-modified mRNA encoding for the spike (S) glycoprotein of SARS-CoV-2 virus [183,203]. This protein is a key component which mediates cell attachment and receptor recognition, allowing viruses to penetrate host cells and cause infection [204]. Phase 3 and 4 clinical trials for both mRNA vaccines have shown high safety, without any significant local or systemic toxicity. A two-dose regime demonstrated that both vaccines are more than 94% effective in preventing serious illness [30,31,205]. It is worth noting that although the PEGylated lipid component is important for improving circulation time, it could be implicated in the allergic reactions observed in some people, and hence, similar mRNA vaccines developed in the future should replace PEG [206,207]. 

Other LNPs–mRNA vaccines have also been tested in animal models against viruses such as Zika [208], Powassan [209], Dengue [210] and Ebola [211], and have shown promising immunisation efficacy.

#### 4.2.3. Inorganic Nanoparticles

Inorganic NPs such as gold, iron oxide and silica have been widely explored as nanocarriers for vaccine delivery because of their low toxicity, biocompatibility and chemical stability (Figure 3C) [132,212,213]. For instance, Chen et al. investigated a vaccine carrier system consisting of gold NPs (AuNPs) conjugated to a synthetic peptide resembling foot-and-mouth disease virus (FMDV) protein [214]. The developed NPs (pFMDV–AuNPs) were evaluated in BALB/c mice, with results showing a three-fold increase in antibody response compared to that of a control system of pFMDV–keyhole limpet hemocyanin (pFMDV–KLH) conjugate. Xu and coworkers prepared surface-engineered Au nanorods as a DNA vaccine adjuvant for HIV treatment, modifying the nanorods with three different molecules: cetyltrimethylammonium bromide (CTAB), poly(diallydimethylammonium chloride) (PDDAC), and polyethyleneimine (PEI) [215]. The results showed that PDDAC- and PEI-modified Au nanorods significantly enhanced cellular and humoral immunity as well as APC activation and T cell proliferation in vivo, in comparison with naked HIV-1 Env plasmid treatment. Niikura et al. examined the effect on immune response of spherical, rod, and cubic shaped Au NPs coated with West Nile virus envelope protein [216]. Researchers observed that rod-shaped NPs were more efficient in macrophage and DCs uptake than spherical or cubic-shaped NPs. Moreover, both spherical and cubic Au NPs induced higher level of inflammatory cytokines, like TNF-α, IL-6, IL-12, and GM-CSF, while rod-shaped Au NPs induced higher secretion of inflammasome-related cytokines, like IL-1β and IL-18. Tao and co-authors reported a system consisting of the extracellular domain of M2 membrane protein (M2e) immobilised on Au NPs and soluble CpG as an adjuvant. This formulation induced high levels of antibody response and provided complete protection against lethal H1N1 influenza virus in a mouse model [217]. In another study, Wang et al. conjugated recombinant trimetric influenza hemagglutinin on Au NPs, coupled with Toll-like receptor 5 (TLR5) agonist flagellin (FliC) as a particulate adjuvant system [218]. IN immunisation in mice with this formulation enhanced influenza-specific IgA and IgG levels and led to antigen-specific IFN-γ secreting CD4^+^ T cell proliferation as well as activated CD8^+^ T cells.

Iron oxide nanoparticles (IONPs) are approved by the FDA for theranostic applications and have been investigated in detail in drug delivery, hyperthermia and magnetic resonance imaging (MRI) as contrast agents for imaging [219,220,221]. IONPs have also shown great potential as a vaccine platform against infectious diseases. Using intravenous route of administration into BALB/c mice, Shen et al. showed that systemic exposure to a single dose of iron oxide nanoparticles loaded with OVA led to subsequent antigen-specific immune reactions. Their studies reported serum production of antigen-specific antibodies lessened as demonstrated by reduction in the serum levels of OVA-specific immunoglobins, IgG1 and IgG2a [222]. A mannosylated nano-vaccine composed of IONPs loaded with HBsAg was more potent than commercial alum-based vaccines in the induction of cellular and humoral immune responses as indicated by studies by Rezaei et al. [223]. In another study, Rybka et al. used superparamagnetic IONPs (SPIONs) as the core of HBV capsid protein self-assembled VLPs, which could facilitate vaccine purification in manufacturing and enhance physicochemical stability [224]. Using two different SPION cores, functionalised with either dihexadecyl phosphate (DHP) or PEG, the authors observed a high efficiency of VLP assembly, particularly with SPION-DHP. However, this strategy also led to a noticeable decrease in antigenicity in comparison with the original antigen, namely at higher DHP and PEG concentrations, which requires further research. 

Silica nanoparticles hold great promise in drug and protein delivery because of their chemical stability, biocompatibility and low toxicity. Moreover, silica NPs can be synthesised in various sizes, shapes and pore diameters. Besides their physicochemical characteristics, these NPs can induce both humoral and cell-mediated immune responses, prompting researchers to investigate their potential as antigen carriers and adjuvants in vaccine delivery [213,225]. Guo et al. investigated the potential of hollow mesoporous silica nanoparticles (HMSNPs) as a vaccine delivery vehicle for Porcine Circovirus Type 2 ORF2 Protein [226]. Researchers studied in vitro uptake and release profiles of protein by HMSNPs, as well as the immune response elicited following IM administration of protein-loaded HMSNPs in female BALB/c mice. The results showed that protein loaded HMSNPs stimulated humoral and cellular immune responses and induced persistent immune responses due to the release kinetics of the HMSNPs. Braun et al. studied the loading and release of VIR-576, a derivative of the natural HIV-1 entry inhibitor targeting the viral gp41 fusion peptide, into/from mesoporous silica nanoparticles (MSNs) in vitro [227]. They demonstrated high peptide loading in the NPs which suggested promise of the formulation for local release applications. However, they recommended further work to be carried out to understand the release kinetics under biological conditions for better translation of in vitro results to in vivo conditions. N4 Pharma has developed Nuvec^®^ Si NPs coupled with polyethyleneimine for the delivery of DNA/RNA antigen into cells. In addition to their antigen delivery role, Si NPs have been reported to show adjuvant effect, generating T helper 1 (Th1) response and high cellular uptake [228]. Theobald suggested Nuvec^®^ as a nonviral vaccine delivery vehicle as a safe and effective alternative to lipid NP systems. It has also been explored as delivery system for SARS-CoV-2 vaccine [228].

### 4.3. Virosomes and Virus-Like Particles (VLPs)

Virosomes and virus like particles (VLPs) have attracted the attention of researchers because of their structural and morphological similarity to infectious viruses, as well as their abilities to bind and penetrate the cell and to stimulate both humoral and cellular immunity. Virosomes are a special type of liposomes consisting of unilamellar mono and bilayered vesicles, to which virus-derived proteins may be attached or inserted [229]. VLPs are composed of a self-assembled viral membrane that forms a monomeric complex [230]. These are empty, multiprotein, nonreplicating and noninfectious structures. Because of the presence of a noninfectious subset of viral components in their structures, VLPs can be considered as a type of subunit vaccine. Both virosomes and VLPs are also safe and stable compared to viral vaccines and soluble antigens [231]. However, virosomes are preferred over VLPs in vaccine delivery. The protein-based structure restricts the movement of VLP while the fluidic phospholipid substrate of virosomes can enhance interactions with host cell receptors [232].

Due to the clinical success of these delivery platforms, several VLP and virosome vaccine products have received market authorization, e.g., for Hepatitis A virus (HAV), marketed as Epaxal^®^ [233,234], Hepatitis B virus (HBV), human papilloma virus (HPV) [232] and influenza (Inflexal^®^) [235]. Both Epaxal^®^ and Inflexal^®^ have been discontinued by Johnson & Johnson in 2011 [236]. Approved HBV vaccines invlude: (i) Heptavax-B, a hematogenous vaccine composed of hepatitis B surface antigen VLP; (ii) Recombivax HB, the first licensed VLP vaccine against HBV, developed by Merck; (iii) Engerix-B, developed by GlaxoSmithKline, which is composed of the viral small envelope protein HBsAg produced in Saccharomyces cerevisiae and presented as particles of around 20 nm size; and (iv) Sci-B-Vac which contains three HBV surface antigens (S, pre-S1 and pre-S2). Furthermore, currently there are three approved prophylactic HPV vaccines in the market, namely Gardasil^®^, Gardasil-9^®^ (a nonvalent HPV VLP vaccine), Cervarix^®^ and Cecolin^®^, all based on L1 major capsid protein self-assembled into VLPs, leading to strong and specific anti-L1 immune responses [134,237].

Inflexal^®^V is an example of a virosome-based approved vaccine, in this case, against trivalent influenza virus. This vaccine is formulated with an inactivated form of two A virus strains and one B virus strain with specific antigen hemagglutinin (HA) and neuraminidase (NA) [235]. This virosome consists of viral lipids, namely phosphatidylcholine, and HA and NA glycoprotein [238]. Inflexal^®^ V has demonstrated excellent humoral immune response against influenza in both adults and children. Epaxal^®^ is another clinically available virosomal vaccine, in this case against HAV [234]. Its virosome consists of phosphatidylcholine and phosphatidylethanolamine with viral envelope antigens, including HA and NA influenza proteins. The virosome surface is decorated with formalin-inactivated HAV which imparts adjuvant properties to the structure. The inactivated HAV triggers B cell proliferation, while the glycoproteins facilitate virosomal uptake by immunocompetent cells, eliciting both humoral and cell-mediated immunity. Bomsel et al. reported the preparation of virosomes containing HA and NA from inactivated H1N1 for the delivery of influenza enveloped viruses, which are used for virosome preparations with added HIV-1 virulence antigens such as recombinant gp41, p1 peptides and 3 m-052 adjuvants. The Gp41 antigen has been shown to aid host cell infection and evoke immune response, leading to full protection of immunised monkeys against vaginal challenge with simian HIV [239]. Virosomes have also been explored for SARS-CoV-2 antigen delivery. SARS-CoV-2 is an enveloped spherical particle with a club-shaped spike glycoprotein expressed on the surface [240]. Specific surface antigens and phospholipids of SARS-CoV2 can thus be used in virosome vaccine production.

### 4.4. Other Advanced Vaccine Delivery Systems

#### 4.4.1. Hydrogels

**Figure 4:**
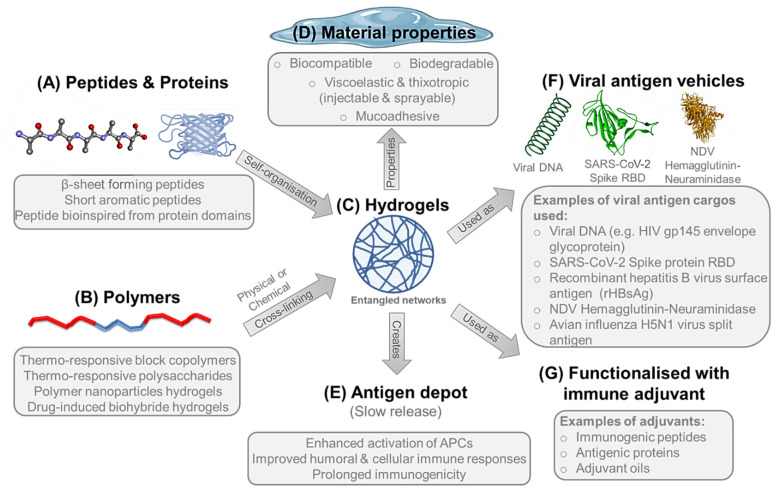
Hydrogels are formed either by the (**A**) self-organisation of peptides or proteins or (**B**) physical or chemical cross-linking of polymers to form (**C**) entangled networks of nanofibrous structures in aqueous matrix, which is (**D**) self-supportive viscoelastic and thixotropic materials that can be injected and sprayed for clinical administration of vaccine formulations. Where natural or bioinspired building blocks are used to create the hydrogel network, the material becomes biocompatible and biodegradable implying low toxicity. Hydrogels are mucoadhesive, so can create localised (**E**) viral antigen depot postinjection/spraying, providing slow and controlled release of antigenic cargo leading to enhanced activation of APCs, improving both humoral and cellular immune responses over a prolonged period. Hydrogels have been used as (**F**) vehicles for various viral antigens and (**G**) could be functionalised with immune adjuvants as stimulatory agents to potentiate the immune response towards the delivered viral antigen.

Supramolecular hydrogels represent an important class of soft biomaterials that has great potential in a wide variety of pharmaceutical and biomedical applications including vaccine delivery. Hydrogels are three dimensional networks of polymeric chains that can retain a large volume of water (>90%) and composed of either high molecular weight natural biopolymers such as polysaccharides and proteins or synthetic polymers and peptides [241,242]. The development of hydrogels with defined material properties can be achieved via molecular assembly of the carefully designed individual monomer molecules. The molecular building units undergo spontaneous molecular recognition and organisation into networks of ordered supramolecular structures with well-defined structural properties, which entangle through either physical or chemical cross-linking forming hydrogels (Figure 4A–C). Physical gels are stabilised by a combination of noncovalent intra- and inter- molecular interactions [243]. These interactions include hydrogen bonding, electrostatic, hydrophobic and aromatic interactions. On the other hand, chemical gels are stabilised by the formation of covalent bonds such as disulphide bonds (through oxidation of thiol groups), photo- or thermal-induced polymerisation, enzyme-catalysed cross-linking, or the reaction between thiols and acrylates or sulphones [244,245,246,247].

##### Peptide Hydrogels

Recently, bioinspired peptide hydrogels have been studied for potential use as vehicles for viral vaccines, thanks to their inherent biocompatibility, biodegradability and mucoadhesion, as well as their viscoelastic and thixotropic properties, implying ease of administration either by injection or spraying, which can be used for both parenteral and mucosal immunisation, respectively (Figure 4A,D) [243,248,249,250]. Besides acting as a vehicle, self-assembling peptide nanofibres can be functionalised with immune adjuvants, such as immunogenic peptide sequences and protein antigens, to modulate immune responses against the corresponding infectious agent (Figure 4F,G) [251,252].

The vaccination of animal models with peptide-based antigen-bearing β-sheet nanofibres resulted in strong immunogenic responses, with activation of both humoral and cellular immunity without the need for any other adjuvants [253]. Grenfell et al. used the synthetic β-sheet forming ionic self-complementary peptide (RADA)4, which underwent self-assembly into nanofibrous hydrogel matrix, as an in vivo depot for the sustained delivery of a recombinant HBsAg (rHBsAg) [253]. Slow-release kinetics of the antigen from hydrogel depot resulted in enhanced activation of APCs with improved humoral and cellular immune responses, leading to prolonged immunogenicity compared to adjuvanted antigens using alum and complete Freund’s adjuvant [253]. Likewise, Friedrich et al. used the β-sheet-forming ionic self-complementary peptide (FKFEFKFE) or (KFE8) that self-assembles into a nanofibrous self-supporting hydrogels as an adjuvant in the development of vaccine against West Nile Virus (WNV) [254]. KFE8 peptide hydrogels emulsified with EIII, the putative receptor-binding domain of the viral envelope protein, were used for subcutaneous vaccination of mice. This system elicited enhanced antibody responses and significant protection against the lethal WNV infection in vivo when compared to EIII delivered with alum as an adjuvant [254]. Peptide hydrogels have also been used as delivery vehicles for viral DNA vaccines. For instance, Tian et al. used the short aromatic peptide Nap-GFFY-NMe, which undergo alkaline phosphatase-triggered self-assembly into nanofibrous hydrogels, for encapsulation and delivery of HIV DNA sequence encoding the gp145 envelope glycoprotein [255]. Enhanced humoral and cellular immune responses were achieved thanks to condensation of DNA by the left-handed structure of nanofibres and thus providing significant protection against degradation, proper transfection, and effective gene expression [255]. 

In a different study, Huang et al. rationally designed the self-assembling peptide sequence FLIVIGSIIGPGGDGPGGD or H9e, bio-inspired from both an elastic domain of spider silk and a transmembrane domain of the L-type calcium channel in human muscles [256]. This peptide showed hydrogel formation in presence of Ca + 2 salts, which was used as an adjuvant for the killed H1N1 influenza vaccine. The results showed improved immunogenicity compared to other commercial adjuvants including oil in water emulsions [256]. The H9e peptide was further evaluated by Li et al. for use as an adjuvant for the modified live vaccines (MLV) of porcine reproductive and respiratory syndrome virus (PRRSV) [257]. Pigs vaccinated with H9e-adjuvanted MLV showed enhanced humoral and cellular immunity governed by the higher number of T helper/memory cells and increased secretion of INF-γ, in comparison to H9e-free MLV [257].

##### Polymeric Hydrogels

Along with peptide hydrogels, polymeric hydrogels have been also introduced as delivery systems for vaccine components (Figure 4B). Roth et al. reported the use of polymer-nanoparticle (PNP) hydrogels as sustained-release delivery systems for both viral antigens and adjuvants to the immune system [258]. Aqueous solutions of both dodecyl-modified hydroxypropylmethylcellulose (HPMC-C12) and poly(ethylene glycol)-b-poly(lactic acid) (PEG-PLA) PNPs were used in combination to form PNP hydrogels rapidly upon mixing. This delivery system was evaluated for immunomodulation using OVA protein and Poly(I:C) which is a toll-like receptor 3 agonist as a model antigen and an adjuvant, respectively. Compared to bolus administration of the same vaccine in standard phosphate buffer saline, the prolonged release of vaccine components from the hydrogel matrix showed enhanced humoral immunity with increased antigen-specific antibody affinity by 1000-folds [258]. More recently, Gale et al. reported the use of the aforementioned injectable (HPMC-C12)-(PEG-PLA) PNP hydrogel for the sustained delivery of vaccine cargo against SARS-CoV-2. The studied cargo was composed of the receptor-binding domain (RBD) of SARS-CoV-2 spike protein as the viral antigen and alum and CpG as adjuvants (Figure 4F). Although RBD is poorly immunogenic even when used in combination with most common adjuvants, the sustained release of the subunit vaccine from the hydrogel matrix increased the RBD-specific antibody (IgG) titres in comparison to bolus administration [259]. 

With recent developments in the design of biohybrid materials responsive to clinically approved small molecules drugs, Gübeli et al. developed a biohybrid hydrogel as a depot system for controlled drug-induced release of HBsAg vaccines, which emerged as a potential replacement to the conventional repetitive vaccination strategy [260]. This system was based on eight-arm branched PEG polymer molecules functionalised with the protein Gyrase B (GyrB), where addition of coumermycin antibiotic induces dimerisation of GyrB, and hence, cross-linking of the polymer, leading to hydrogel formation and encapsulation of vaccine cargo within the gel matrix [260]. Orally administered novobiocin acted as a molecular switch to the hydrogel matrix by competitively replacing coumermycin, unlocking GyrB dimers, dissolving the hydrogel and releasing the vaccine. The novel depot system elicited a comparative immune response to that of the repetitive regime [260].

Thermo-responsive polymers that form hydrogels at body temperature also proved to be useful for formulation of vaccine depot gels. An example of this is the thermosensitive triblock copolymer hydrogel comprised of (PLGA-PEG-PLGA), which was utilised by Gao et al. to develop a DNA vaccine delivery system for the encapsulation of the recombinant hemagglutinin-neuraminidase plasmid of the avian Newcastle disease virus (NDV) [261]. This triblock copolymer undergoes postinjection hydrogelation triggered by host body temperature, leading to sustained release of the plasmid from the hydrogel matrix. The vaccine triggered strong immune responses, high efficacy, and complete protection against highly virulent strains of NDV [261].

Furthermore, thermo-responsive hydrogels generated from polysaccharides-based polymers have been also used for viral vaccines delivery. Wu et al. utilised the quaternised chitosan derivative N-[(2-hydroxy-3-trimethylammonium) propyl] chitosan chloride (HTCC) in combination with α, β-glycerophosphate (α, β-GP), HTCC/GP, for the development of a thermo-sensitive hydrogel intended for IN delivery of the avian influenza H5N1 virus split antigen [262]. At body temperature, the intranasally-administered system showed sol-gel transition, leading to prolongation of the antigen residence time in the nasal cavity. The enhanced humoral and cellular immune responses and the increased antigen-specific mucosal IgA titres elicited by the adjuvant-free H5N1 hydrogel vaccine, when compared to the naked H5N1 split antigen and MF59 adjuvant/antigen complex, were all attributed to prolonged release of antigen and disorganisation of ZO-1 protein of the nasal epithelium resulting in enhanced transepithelial transport of the antigen [262,263]. Moreover, this HTCC/GP thermosensitive hydrogel vaccine delivery system was previously used for IN vaccination with the adenovirus-based Zaire Ebola virus glycoprotein antigen (Ad-GPZ) [264]. Serum IgG, IgG1, and IgG2a and mucosal IgA antibodies had the highest titres in response to the hydrogel-based vaccine due to prolonged residence time of the antigen in the nasal cavity [264].

#### 4.4.2. Microneedles

In the pursuit of innovative administration routes for vaccines, the skin has emerged as an interesting alternative to conventional parenteral routes. This is mainly due to the extensiveness of this organ, and the easy access to immune cells, which abundantly populate the dermis. For this reason, achieving antigen and adjuvant delivery to this region has increased the number of possibilities of generating efficient local and systemic immune responses [265]. However, the external surface of the skin (the stratum corneum) is a very strong and impermeable barrier, making it extremely difficult for conventional drug and vaccine formulations to cross it and reach the dermis. This has led to the development of various strategies to disrupt this barrier and access the dermal space, including chemical and physical methods such as the use of penetration enhancing molecules, iontophoresis, electroporation and microneedle (MN) arrays [266]. This latter strategy has shown particular promise in vaccine delivery, with some prototypes achieving early stages of clinical development.

Microneedle arrays are composed of tens to hundreds of needle-shaped projections, usually shorter than 1 mm, in various shapes and geometric arrangements. Over the years, these devices have been manufactured in a variety of materials including metals, glass, ceramics and polymers, through different methods including injection moulding, solvent casting, laser micromachining, drawing lithography and, more recently, three-dimensional (3D) printing [266,267]. The different types of MN arrays have traditionally been classified as solid, coated, hollow, dissolving and hydrogel-forming.

**Figure 5:**
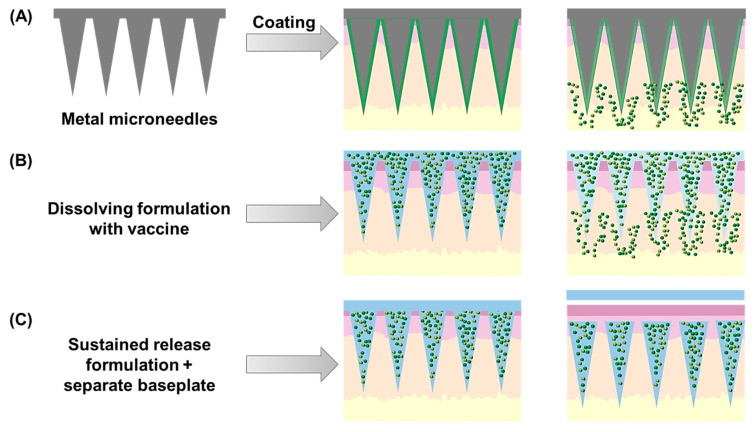
Schematic representation of the three main types of microneedle arrays developed for vaccine delivery. Coated microneedle arrays (**A**) are prepared using a solid array base, usually metallic, which is coated with a dissolving formulation containing the antigen(s) and adjuvant(s). Alternatively, dissolving formulations (**B**) can be used to manufacture the entire array, leading to vaccine delivery upon skin insertion of these self-disposable devices. Finally, sustained-release formulations (**C**) have been used to produce implantable microneedle tips that are left in the skin after insertion, upon removal of a separate baseplate.

Solid MNs were the first to be developed, aiming at a “poke and patch” approach in which drug permeation from a patch is improved by the transient pores created by the MNs in the skin [268]. From this concept, researchers developed coated and hollow MN arrays, aiming at improving the delivery efficacy of these devices. In the case of coated arrays, the drug or vaccine is directly coated onto a solid MN array, releasing within the skin upon insertion. On the other hand, hollow MN arrays mimic hypodermic needles through incorporating a channel within the needle shafts for delivery of liquids to the dermal space [269]. More recently, dissolving and hydrogel-forming polymer-based MN arrays have been developed from biodegradable polymers, allowing the achievement of self-disposable systems. This presents several advantages, particularly in terms of waste management and reduced risk of needle-stick injuries [270]. Moreover, this strategy also allows the delivery of increased doses of drugs and antigens, either incorporated in the needle shafts or as part of external reservoirs dissolved by the interstitial fluid absorbed upon MN array insertion [271]. Figure 5 summarises the three types of microneedle arrays developed and evaluated in the last few years for viral vaccination purposes, described in the following subsections.

##### Coated MN Arrays

Considering the low antigen doses commonly administered in vaccines, coated MN arrays were initially chosen for this application, with promising results obtained by the Prausnitz group in the scope of influenza immunisation. Using stainless steel MN arrays, the group successfully coated various inactivated influenza virus strains onto these devices, using carboxymethyl cellulose (CMC), Pluronic F-68 and trehalose as additional excipients [272,273,274,275,276]. In general, these studies showed that TD immunisation of mice led to strong humoral and cellular immune responses, providing protection against challenge, at least as effectively as IM immunisation with the same antigens. Effective viral clearance from mice lungs, as well as induction of memory responses, were also achieved with the developed coated MN array systems. The same coating strategy was later applied by the same group for the TD delivery of a plasmid DNA encoding hepatitis C virus nonstructural 3/4A protein [277]. In this study, MN-based immunisation effectively elicited specific cytotoxic T cell responses in mice, in similar levels to those generated following gene gun-based cutaneous administration. Stainless steel MN arrays were more recently used by Seok et al., who used a polyvinylpyrrolidone (PVP) coating solution containing trehalose to deliver polyplexes containing PLGA nanoparticles, polyethyleneimine and a DNA H1N1 influenza vaccine [278]. Despite achieving enhanced IgG-based immune responses with the polyplex-coated MN arrays in comparison with pDNA-coated ones, the expression level of exogenous genes was low, resulting in low immunogenicity of the vaccine prototype.

On the other hand, the Kendall group developed a different coated MN device for vaccination purposes, obtaining similarly promising results in various viral vaccines. The device, named Nanopatch™, consists of a densely packed array of very short silicon MN (100 µm in length) and was successfully coated by the researchers with a commercial seasonal trivalent influenza vaccine (Fluvax^®^ 2008) [279]. Applying two devices to each mouse, the authors observed a 100-fold dose reduction in comparison with IM immunisation with the same vaccine, leading to high and long-lasting antibody responses. This approach was further expanded to other viral vaccines in different presentations, from HPV virus-like particles [280], to antigen-encoding DNA targeting West Nile virus, Chikungunya virus [281] and herpes simplex virus [282,283]. In further studies, the authors optimised the formulation to achieve higher antigen delivery [284], to include an adjuvant and achieve synergistic immune response improvements [285], and to assess vaccine kinetics to peak serum antibody levels in comparison with IM injection [286].

Another type of coated MN arrays for immunisation are those made of polylactic acid (PLA). Nguyen et al. described coating of these arrays with HBsAg in a CMC gel solution, with or without trehalose as a stabiliser [287]. Mice immunised with two doses of the MN prototype elicited higher antibody responses than those receiving the same antigen through IM route, with a Th2-biased response. Moreover, the inclusion of trehalose in the formulation increased antigen stability at 40 °C up to 7 days and after 10 freeze-thaw cycles. A similar strategy was recently described by Choi et al., i.e., coating a live smallpox vaccine in a PVA and trehalose solution to PLA MN arrays [288]. This approach not only provided improved stability to the vaccine in storage at −20 °C, but also led to increasing antibody titres up to 12 weeks postimmunisation. On the other hand, Uppu et al. described the application of coated PLA MN arrays in an immunisation strategy against dengue virus, through layer-by-layer coating with different polymers and adjuvants [289]. The results showed vaccine uptake by immune cells in both mice and human skin, with antigen release kinetically controlled by the degradation of the polymers used in the layer-by-layer coating. Finally, Jeong et al. proposed an innovative device with two semicircles of PLA MNs independently coated with two different influenza vaccines in a CMC and trehalose solution [290]. With this approach, the authors achieved equivalent immunisation efficacy to that of separately administered coated MN vaccines and a mixture of both vaccines coated onto a single MN array. Additionally, mice survival rate after viral challenge was equivalent or higher in the group immunised with the compartmental MN array in comparison with mice receiving the vaccine mix coated onto a single MN array.

##### Dissolving MN Arrays

Despite the success of coated MN-based approaches in immunisation, other strategies have been developed to overcome the limitations of potentially reusable devices, including the risk of needle-stick injuries and the need for appropriate disposal of the solid MN arrays after use. Polymeric MN arrays are particularly well-suited for this purpose, as they can be manufactured using biodegradable polymers, rendering self-disposable devices [270]. Dissolving MN arrays showed particular promise in the vaccine delivery field, allowing the incorporation of the vaccine antigen and adjuvant within the MN matrix, fabricated from fast dissolving polymers. Upon skin insertion, these MN arrays come in contact with interstitial fluid and quickly dissolve, releasing the vaccine in the epidermis and dermis where it can access the abundant resident immune cell populations. In the development of dissolving MN vaccine delivery systems, it is important to consider not only manufacturing and scale-up aspects, but also the various factors that affect the immunogenicity and efficacy of these approaches, including polymer selection, formulation pH, array geometry and needle density [291].

In 2010, the Prausnitz group reported for the first time the use of dissolving MN arrays for immunisation against influenza [292]. Here, the authors reported the fabrication of PVP MN arrays encapsulating a freeze-dried form of an inactivated influenza virus vaccine. These MNs dissolved quickly in mice skin, delivering more than 80% of the antigen in 15 min. Moreover, single-dose immunisation of mice with dissolving MN arrays induced strong humoral and cellular immune responses, in levels at least comparable to those achieved with IM immunisation and leading to effective protection against lethal viral challenge. In the same year, the Kendall group also published their first report of dissolving MNs in influenza vaccine delivery, using the previously described Nanopatch™ technology [293]. These multilayered MNs, composed of CMC and loaded with the commercial influenza vaccine Fluvax^®^ 2008, were able to elicit potent antibody responses which persisted in time, which is a sign of efficient memory induction.

After these initial proof-of-concept studies, numerous other publications reported the development, manufacturing and evaluation of dissolving MNs for vaccination [270]. In terms of viral vaccines, influenza has been the main focus of attention. In 2012, Matsuo et al. reported the development of hyaluronan-based dissolving MN arrays for the delivery of various antigens including hemagglutinin specific to three influenza strains [294]. The results showed that transdermal immunisation of mice generated strong and long-lasting antibody responses, comparable to those achieved with IM injection and higher to the ones obtained by ID or IN immunisation, regardless of the presence of adjuvants in the formulations given through these other administration routes. Moreover, the MN-based immunisation strategy also provided protection against challenge, similar to that achieved through IM and IN vaccination. Similar results were obtained by Kommareddy et al., who used CMC-based MN arrays and monovalent H1N1 or trivalent influenza antigens [295]. The authors further demonstrated that a prime-boost TD immunisation regimen could generate antibody responses stronger than those obtained with IM injection. Research in this field continued with various authors demonstrating the efficacy of polymeric MN arrays for influenza immunisation, particularly using dextran [296], gelatine [297], polyvinyl alcohol (PVA) [298], hydroxyethyl starch [299] and CMC [300,301]. More recently, Vassilieva et al. additionally demonstrated the potential of dissolving MN arrays to codeliver influenza antigens and adjuvants such as Quil-A saponin or cGAMP, with promising results particularly for older populations [302]. Similarly, Wang et al. reported the efficacy of MN-delivered vaccine nanoparticles containing influenza matrix protein 2 (M2) ectodomain (M2e), neuraminidase and the adjuvant flagellin [303]. The results evidenced strong and protective humoral and cellular immune responses against homologous and heterosubtypic influenza viruses with this approach, paving the way to a universal influenza vaccine.

Some of these approaches reached clinical development with promising results. Hirobe et al. developed MN arrays composed of hyaluronan, dextran and povidone to deliver trivalent hemagglutinin antigens transdermally [304]. The MN arrays were administered twice to healthy men (20 to 49 years old) and elicited an effective immune response at half the dose required for SC administration, without generating any noticeable systemic adverse effects. More recently, Rouphael et al. reported the results of a Phase 1 trial on the safety, immunogenicity and acceptability of gelatine MN arrays loaded with hemagglutinin antigens against three influenza strains (H1N1, H3N2 and B) [305]. In this work, TD immunisation with dissolving MN arrays led to similar antibody titres as those observed with IM immunisation, regardless of whether the MNs were applied by a healthcare professional or self-applied by the participants. Importantly, lower pain scores were reported by the participants in comparison with IM injection, and a general preference for TD vaccination was registered in this study. The authors also recently published an additional analysis of the results obtained in this study, particularly in terms of the mechanisms behind the immune response observed in the different study groups [306]. The results showed that hemagglutinin inhibition titres and antibody avidity were similar between TD and IM immunisation, despite the lower antigen dose in the MN array group. MNs also induced higher neuraminidase inhibition titres and T follicular helper cell levels, confirming an overall response that was at least equal to IM vaccination.

Despite the main focus on influenza, other efforts have looked at dissolving MN arrays for vaccines against polio, measles, HIV, and other viruses. For example, the Prausnitz group expanded the evaluation of PVA-based MN arrays to the delivery of a rabies DNA vaccine for dogs [307] and of a Zika virus inactivated particle [308]. In both studies, immunisation with dissolving MN arrays elicited strong humoral and cellular immune responses, at least comparable to those obtained with IM injection, with low antigen doses. Moreover, in the case of the Zika virus vaccination, the MN-based approach led to cross-protection against different Zika virus strains and also dengue virus serotypes, efficiently controlling viral titres and inflammatory reactions. PVA-based MN arrays were also evaluated by Donadei et al. for the delivery of an inactivated polio vaccine, achieving high specific IgG responses with lower vaccine doses than those administered intramuscularly [309]. Similarly, Edens et al. reported the use of dissolving MN arrays for the delivery of inactivated polio vaccine [310] and a measles vaccine [311] to rhesus macaques. In these studies, results showed the induction of neutralising antibody titres against both viruses, comparable to conventional immunisation routes such as SC and IM. A combined approach against measles and rubella was also described by Joyce et al., who used CMC-based MN arrays [312]. In this case, TD immunisation elicited protective antibody titres against both viruses at higher levels than SC injection and protected the animals from viral challenge with wild-type measles. It is worth mentioning as well the results obtained by Zhu et al. in MN-based immunisation against enterovirus 71 (EV71), the causing agent of hand-foot-and-mouth disease [313]. Here, the authors used hyaluronan MN arrays to deliver EV71 virus-like particles through the skin, achieving robust and protective immune responses at a 10-fold lower antigen dose in comparison with conventional IM vaccination.

Hepatitis B and HIV have also been the focus of dissolving MN vaccine development. In 2012, Pattani et al. described the development of Gantrez^®^ MNs loaded with a recombinant HIV antigen (gp140) and monophosphoryl lipid A as an adjuvant for TD immunisation [314]. Mice received a total of four vaccine doses (days 0, 14, 28 and 42) in different combinations of administration routes: MN prime and intravaginal boosts, MN prime and IN boosts, all SC injections or all MN administrations. The developed MN arrays were able to prime antigen-specific IgG responses, which increased particularly with IN boosts. This immunisation regimen led to increased serum and mucosal antibody levels, at least similar to those elicited by SC injection, and higher in the case of IgA. Other studies reported the use of dissolving MN arrays to deliver a recombinant human adenovirus type 5 vector encoding HIV-1 gag protein [315,316]. In this case, MN-based immunisation led to potent cytolytic multifunctional CD8^+^ T cell responses in mice, promoted by a specific subset of DCs present in the skin. The authors also demonstrated that this cellular response was long-lived and retained recall capacity for memory responses up to two years after immunisation. In the case of hepatitis B virus, Qiu et al. described the use of PVP MN arrays for the TD delivery of a plasmid DNA vaccine with or without additional adjuvants such as CpG ODN, cationic liposomes or both [317]. High antibody responses were observed in this immunisation approach, particularly when the antigen was encapsulated in the cationic liposomes and administered with CpG ODN. On the other hand, Perez Cuevas et al. reported the use of CMC MN arrays for HBsAg delivery to mice and rhesus macaques [318]. These MN arrays elicited antibody responses comparable to those obtained with IM immunisation, without any additional adjuvants. More recently, Kim et al. presented a combinatorial approach consisting of a PLA/CMC MN tip loaded with HBsAg for slow release and an antigen-loaded CMC coating for bolus release [319]. The results showed an effective immune priming by the bolus antigen release from the CMC coating, followed by a boost effect generated by the slow antigen release from the PLA MN tips.

Finally, it is worth noting the role played by this type of MN arrays in the endeavours to vaccinate against SARS-CoV-2. In early 2020, Kim et al. reported the use of CMC MN arrays for TD immunisation with recombinant viral proteins from MERS and SARS-CoV-2 virus [320]. In this study, results showed substantial increases in specific antibody levels at two weeks postimmunisation with MN arrays, in comparison with earlier time points. Similarly, Kuwentrai et al. described the delivery of SARS-CoV-2 spike protein’s RBD using hyaluronan MN arrays, and including alum as an additional adjuvant [321]. This approach elicited high and long-lasting antibody responses, as well as significant T-cell responses, measured by interferon-gamma (IFNγ) expression. Interestingly, these results were not achieved when the same system was used to deliver mRNA, in an attempt to simulate current SARS-CoV-2 vaccines based on this technology.

##### Implantable MN Arrays

In recent years, a new type of biodegradable MN arrays has been studied for long-acting drug delivery and, in a few studies, for vaccination purposes. Implantable MN arrays usually consist of slowly degradable needle tips loaded with the antigen or drug of interest and supported by a fast-dissolving backing, which allows implantation of the needle tips inside the skin upon application. In vaccine delivery, this approach is particularly interesting to control vaccine kinetics and antigen delivery to the lymphatics, which can greatly influence the immune response elicited [41]. Chen et al. described the development of an implantable array with chitosan needle tips containing an inactivated influenza vaccine and a fast-dissolving PVA/PVP backing layer [322]. The results showed higher antibody levels in the MN group than those observed in the IM immunisation group, a fact the authors attributed to the adjuvant activity and depot effect of the chitosan needle tips. Moreover, MN vaccination led to efficient protection of mice against viral challenge, in comparison with conventional IM injection. In another study, Boopathy et al. reported the enhancement of humoral immune response against an HIV trimer antigen by vaccination with an implantable MN array [323]. The authors used in this case silk fibroin protein to form the antigen-loaded needle tips, which elicited sustained release in the skin over two weeks. This allowed not only an increased retention of the vaccine in the administration site, but also higher colocalisation of the antigen with follicular DCs in the draining lymph nodes and increased priming of germinal centre B cells, essential in the development of long-lasting antibody responses. One month after vaccine administration, the MN-immunised group showed 1300-fold higher antibody levels than the group receiving a single-dose intradermal injection of the same vaccine, demonstrating the potential of this approach for HIV vaccination.

Despite these promising results and the demonstrated potential for MN-based vaccination, it is worth considering some challenges concerning the development of these products at clinical level until market approval. In terms of product development and manufacturing, researchers should consider minimising the number of process steps to facilitate up-scaling and high-quality GMP manufacturing, as well as other possible requirements such as aseptic fabrication, sterilisation at the endpoint and costs of production [53]. Additionally, common vaccine-related issues such as stability in storage and cold-chain requirements must also be taken into consideration at this stage [34]. Achieving stable vaccine formulations in MN array format, which can be stored at room temperature and withstand high temperatures characteristic to certain climates, could be a game-changer in terms of worldwide vaccine coverage and distribution [324].

## 5. Clinically Approved Nanovaccines against Viruses

Generally, development of vaccines must go through different stages of preclinical and clinical testing in order to gain approval for production and marketing. The first stage of vaccine development journey is the preclinical stage in which the infectious agent is extensively investigated for immunogenic antigens that can trigger immune responses in the host. Outcome of preclinical studies is assessed by the regulator, which will authorise the developer to start clinical trials only if the benefit of the developed vaccine outweighs risks of undesired side effects or toxicity. Clinical trials involve studying the effects of vaccines under development in human subjects over three sequential phases. Phase 1 involves vaccine testing in a small group of healthy adult volunteers to ensure that the developed product is free from major safety concerns and to evaluate dose-ranging and the elicited immune response. Phase 2 trials involve a pilot efficacy study for a larger group of volunteers and to confirm safety. If the vaccine under investigation demonstrates efficacy and low risk of general toxicity, it will enter the third phase of clinical trials. Phase 3 trials involve a much larger group of a wider population range, often tens of thousands of people, involving volunteers from different areas of high viral transmission rates, elderly people, and those with underlying health conditions, in order to confirm safety in these groups, efficacy, and the effective dosing level of vaccine. Successful vaccines from Phase 3 can seek marketing authorisation from regulatory authorities for mass production and marketing. After marketing authorisation, vaccine products will enter Phase 4 of pharmacovigilance, in which they will be continuously and carefully monitored for safety and efficacy [325,326]. In emergencies as in pandemics like the current COVID-19, a vaccine cannot be fully approved and can be developed under “emergency use authorisation” to facilitate its availability and use for mass immunisation, even if it is still under clinical trials [326,327]. There are currently about 320 vaccine products against SARS-CoV-2 under development, with approximately 126 vaccines in clinical trials and 194 in the preclinical assessment [328]. However, only eight candidates are in Phase 4 clinical trials after being developed and marketed to assess their performance in real life scenarios and to detect the long-term effects in the general population [326,328]. Of these eight, only BNT162b2 and mRNA-1273 of Pfizer-BioNTech and Moderna, respectively, are based on LNPs as nonviral vector nanocarriers (Table 1).

In spite of the limitations associated with traditional vaccines using live-attenuated or inactivated viruses, such as the time-consuming manufacturing process, toxicity and high infectivity, there are, at present, a limited number of fully approved vaccines against viruses utilising an advanced biomaterial or nonviral nanocarriers for their development and delivery [328,329]. Historically, virosomes and VLP systems showed clinical success for both HAV and HBV vaccines [233,234], as well as influenza [235,238], and more recently with the prophylactic HPV vaccines [134,237] (Table 1). However, the herpes zoster subunit vaccine [HZ/su], Shingrix^®^, developed by GlaxoSmithKline, is one of a few nanovaccines approved for clinical administration, which uses liposomes for the delivery of viral antigen cargo (Table 1) [190,191,192]. Therefore, there is still a lot of work to be done for the development of clinically approved nanovaccines, which can satisfy the stringent quality, safety and efficacy requirements of regulators. The new generation of LNP-based SARS-CoV-2 vaccines, which are under Phase 4 clinical evaluation, has opened the door to advanced nanotechnological approaches for the development of other nanovaccines (Table 1) [328,330,331]. LNPs of both BNT162b2 and mRNA-1273, were utilised to encapsulate and deliver the nucleoside-modified mRNA encoding the full-length spike (S) glycoprotein of SARS-CoV-2 virus (Table 1) [330,331]. These vaccines elicited strong humoral and cell-mediated immune responses to the S antigen protecting the host against SARS-CoV-2. The main disadvantage of these vaccines is stability, which requires storage at ultralow temperature from −80 to −60 °C and from −50 to −15 °C, for Pfizer-BioNTech and Moderna vaccines, respectively [330,331]. Also, Pfizer-BioNTech vaccine can be stored frozen at −25 to −15 °C for two weeks only, requiring special transport and storage equipment [330]. On the other hand, Moderna vaccine can be stored refrigerated at 2–8 °C for 30 days [331]. Other SARS-CoV-2 LNP-based vaccines include Cov2 SAM (LNP) vaccine (GlaxoSmithKline, Phase 1 clinical trial), LNP-nCoV saRNA (Imperial College London, Phase 1), LNP-nCOV saRNA-02 vaccine (MRC/UVRI and LSHTM Uganda Research Unit, Phase 1), and HDT-301 vaccine (SENAI CIMATEC, Phase 1) which comprise self-amplifying RNA (saRNA) encapsulated within LNPs, and are all still in early development stages [328].

In addition to the use of nanotechnological approaches in vaccines delivery, other advanced techniques such as electroporation has been used for intracellular delivery of the intra-dermally injected INO-4800 vaccine (Inovio Pharmaceuticals (San Diego, CA, USA)/International Vaccine Institute (Seoul, Korea)/Advaccine Biopharmaceuticals Suzhou Co., Ltd. (Suzhou, China), Phase 2/3 clinical trials) [328,332,333]. INO-4800 is a DNA vaccine which contains the plasmid pGX9501 encoding SARS-CoV-2 full length Spike glycoprotein. This vaccine utilises a small electric pulse generated from a hand-held smart device to reversibly make small pores in the cell membrane, promoting plasmid cellular transfection and activating immunotherapy. The vaccine was reported to be immunogenic, generating robust humoral and cellular immune responses, and was shown to be safe and well-tolerated [332,333]. Compared to other SARS-CoV-2 vaccines, INO-4800 was reported being stable at room temperature for more than a year, not requiring special freezing conditions during shipment and storage [333]. Other examples of electroporation-based vaccines under clinical investigation include the VGX-3100 synthetic DNA vaccine targeting HPV16/18 (Phase 2b trial) [334], a CMV DNA vaccine (Phase 2 trial) [335] and few others in Phase 1 trial reviewed by Xu et al. [336]. 

Microneedle-based technologies for TD vaccination are also under clinical development at the moment. For instance, a Phase 1/2 double-blind randomised trial sponsored by Micron Biomedical, Inc. (Atlanta, GA, USA) currently recruiting participants (NCT04394689), in which the safety and immunogenicity of a measles-rubella dissolving MN array vaccine will be evaluated in adults, toddlers and infants in comparison with SC administration of a WHO prequalified vaccine [337]. Previous studies have also demonstrated the efficacy of MN-based vaccines at the clinical level, particularly with an influenza vaccine coated MN array developed at Georgia Institute of Technology by the Prausnitz group [305]. 

The use of nanomaterials and other advanced technologies is indeed a potential approach for the effective and safe delivery of vaccines based on viral genetic materials, which is currently being rigorously tested for SARS-CoV-2, but could be generally applied to other vaccines in the near future. However, there are emerging challenges for the regulatory clinical and marketing authorisation of nanomaterials-based vaccines that need to be considered from early stages of development, which are discussed in the following section.

## 6. Manufacturing Consideration and Regulatory Requirements for Pharmaceutical Development of Nanovaccines

A vaccine product is often engineered with four main components: an immunogen that can induce an immune response derived from the pathogen (proteins, peptides, lipids, mRNA); adjuvants, which are stimulatory agents that potentiate the immune response towards the delivered immunogen (independent or as a conjugate to the immunogen); delivery strategy, which utilises nanocarriers to encapsulate or present the immunogen to APCs in a stable and targeted manner (for instance, viral vectors, nanocarriers or hydrogels); and finally, a device designed to physically administer the vaccines (syringes, implants, microneedle patches) [29,329,338]. There are various quality, safety and efficacy regulatory requirements for the development and production of these complex biopharmaceutical products, from early stages of development, passing through the various stages of manufacturing, ending by storage and distribution. Here, we will shed the light on these regulatory requirements, mainly for the upstream and downstream processing, highlighting special considerations for nanovaccines formulation development. 

### 6.1. Upstream Processing

The manufacturing process of nanovaccines can be considered identical to that of conventional vaccines for upstream processing. Once the fabrication method of the immunogen has been selected (live-attenuated, Inactivated/killed, subunit, conjugation, recombinant, recombinant vector or VLP), the Master Viral Seed (MVS) can be produced and extensively characterised to ensure purity and safety [339]. Depending on the starting material, primary cell lines, continuous cell lines or chicken eggs can be used as substrate to grow the virus. These production platforms each have their own advantages and drawbacks. Thus, during development and selection, the following points should be considered: yields, effects of post-translational modifications, cost, contamination risks, scalability, complexity, production timescale, glycosylation profiles and frameworks for regulatory approval [340,341,342,343,344]. 

From this initial bank, Working Viral Seeds (WVS) are propagated for production lots to ensure consistency and confidence in the quality of the final product. WVS can be considered intermediate products, as multiple strains or different subunits can be blended in the final bulk during formulation. The use of master and working seed lots provides a method to limit the replication of the seed and to minimise the possibility of genetic variation.

This is followed by the isolation of the immunogen from its environment, generally via centrifugation and homogenisation [345]. Further manipulations of the material, including encapsulation into a nanocarrier can be considered as downstream processing as the immunogen is unaltered after this point. 

### 6.2. Downstream Processing

Once the immunogen is in its free form, the material can be purified using one of the following methods: sterile filtration [346], solvent extraction [347], alcohol precipitation [348], ultrafiltration [349], gel permeation chromatography [350], zonal centrifugation [351], formaldehyde inactivation [352], diafiltration [353], detergent precipitation [354] and various other methods of chromatography. Depending on the selected nanocarrier delivery strategy an appropriate encapsulation method is utilised. For liposomal manufacturing, the three basic techniques include: (1) mechanical methods such as film hydration and microfluidisation, (2) solvent displacement such as ethanol injection and reverse phase evaporation and (3) detergent depletion methods [355]. Several other methods can be used for the production and incorporation of the immunogen; however the following points should be considered during the selection process:

- Scalability—small scale laboratory research should have the capability to be easily scaled to meet market requirements, taking into account technological limitations;
- Use of organic solvents—most recognised methods use organic solvents; however, due to their detrimental effect on health, they need to be limited to minor amounts of class II solvents such as chloroform and methanol to meet European and US pharma-copeial requirements;
- Consistency—As the utilisation of nanocarriers increases the surface area this has an effect on the biodistribution profile leading to unpredictable reactivity. To limit the chances of any unwanted reactivity it would be important to characterise and control the physicochemical properties (size distribution, charge, lamellarity, entrapment efficacy, phase transition temperature, antigen release profile) between batches;
- Temperature—Most immunogens are only stable at lower temperatures; hence, any methods that require higher temperatures cannot be utilised.

Once the immunogen has been loaded into its nanocarrier, size manipulation is often carried out using homogenisation, which applies shear forces to achieve uniform size distribution [356]. These factors are often controlled using the pore size of the filter and the number or recirculation cycles.

At this point, intermediate products can be stored at low temperatures, depending on the results from their stability testing. Hence, a point to consider during the validation and qualification of the vaccine and the method of manufacture should be the stability of the nanocarriers at different temperatures over various time points. For both the intermediates and the final bulk, stability tests are required for physicochemical analysis and biological assays. The implementation of a stability protocol should be based on detailed information about the types of testing, including specifications, testing intervals and data analysis.

### 6.3. Formulation Considerations

Suitable controlled quantities of all ingredients are blended to uniformity to produce the final bulk, this may include; buffers, bulking agents, stabilising excipients, adjuvants and preservatives. As with the development of any pharmaceutical product, the addition and concentration of each excipient needs to be meticulously justified to the regulators and sufficient relevant data should be provided. If the intermediate production had a sterile filtration step during downstream processing the bulk would need to be prepared aseptically, otherwise a sterilisation step is required at the end of processing.

If the vaccine product is to be commercialised as multistrain, multiple intermediates containing different WVS can be blended. However, before the blending process can begin, the intermediate endotoxin and potency should be tested. The most commonly recognised tests in industry are Limulus Amebocyte Lysate (LAL) for endotoxin evaluation [357] and plaque formation assays, endpoint dilution assays (tissue culture infective dose TCID_50_) virus neutralisation assays or quantitative polymerase chain reaction (PCR) assays for potency [358]. The purpose of this evaluation before the blending process is that intermediates with high potencies or endotoxin can be blended with intermediates with low potencies or endotoxin to meet the specification set out in the license. The effect of the nanocarriers should be considered during product development for these specifications, as they may mask the accuracy of the result. Most nanocarriers exhibit the following three-phased release profile: (1) burst release due to the desorption of molecules attached to their surface; (2) an intermediate phase which is released as the matrix of the carrier degrades; and (3) the final release of the encapsulated material [359]. Hence, the release profile should be accurate characterised to precisely design a potency and endotoxin assay.

During formulation development the interactions of the components should also be meticulously studied to design nanovaccines with optimum activity. These interactions include, but are not limited to, immunogen-nanocarrier interactions and adjuvant-nanocarrier interactions. Immunogen-nanocarrier interactions could include the altered antigen presentation to APCs which can either be enhanced or inhibited. For example, poly(vinylalcohol)-coated iron oxide nanoparticles have been shown to inhibit the processing of OVA by DCs to stimulate CD4^+^ T-cells [360], whereas, poly(d,l-lactic-*co*-glycoside)-based polymeric nanoparticles significantly improve antigen presentation and T cell activation by OVA in DCs [361]. Adjuvant-nanocarrier interactions should also be extensively studied to avoid any hyperactivation of the adjuvant in the presence of the nanocarrier. Several vaccines have been known to be recalled from the market due to adjuvant toxicity resulting in the stimulation of CD28 on T cells triggering hypercytokinemia [362].

Furthermore, as nanocarriers could have unpredictable biological activities in a host per se, considerations should be taken to study their effect in clinical studies compared to the bulk material. The major nanotoxicity pathways in cells could involve oxidative stress [363,364,365], inflammation [366,367,368] and genotoxicity [369,370,371], which therefore requires extensive preclinical and clinical toxicity assessment of nanocarriers used for vaccine formulations.

### 6.4. Quality Control and Release Testing

As with any other biopharmaceutical product, manufacturers of nanovaccines are bound to perform appropriate tests for the licensed specification according to 21 CFR 610 for the US markets [372]. For nanotechnology enabled products these specifications are assessed on a case-by-case basis due to the lack of uniformity between regulators. In general, the release lots of the final product must meet the standards of safety, purity and potency established for the particular vaccine product highlighted in ICH Q5C [373]. Examples of these test include, but are not limited to potency assays, general safety (detection of adventitious agents), sterility, bacterial endotoxin, purity, residual moisture, pyrogen, identity and constituted materials. Samples should be taken throughout the manufacturing process to maintain and document quality control of the processed batch. However, for general safety and bioburden testing, the samples should be taken at the “dirtiest” step of the manufacturing process to demonstrate the absence of contaminants in the product.

### 6.5. Regulatory Requirement and Challenges

Although it has been over 15 years since the first protein-based nanoparticle drug Abraxane^®^ (albumin-bound paclitaxel) was approved for use in 2005 by the FDA [374], nanomedicines continue to challenge the regulatory authorities. The main role of the regulator is to ensure quality, safety and efficacy of all medical products and devices through existing, well-defined regulatory frameworks; however, as both the scientific innovations and market expectations evolve, it is becoming increasingly difficult to set specific guidelines.

A lack of guidelines and harmonisation from these regulatory bodies is causing a high degree of uncertainty for product developers, hindering the development and marketing of novel nanotechnology-enabled products. To ensure a smooth approval process, the identification of, and a general agreement on, the regulatory requirements applicable for the evaluated product/device are therefore prerequisites. Thus, nanotechnology enabled products are often developed and scaled-up with involvement from the authorities.

Utilising current regulatory frameworks, the approval process involves the requirement of four principle elements outlined in Table 2. Generally, regulators require release and stability data from pilot batches, which are prepared using the same process as that of the intended product for the market. In addition, they also require excipient information, detailed rationale of the manufacturing process (purification, sterilisation … etc.) and validation methods for the release tests used including a justification for their use. A justification should also be provided for the nanocarrier utilised in conjunction with preclinical data established in an animal model characterising the immune response for each of the component parts (adjuvants/nanocarriers) as well as the final vaccine composition. In addition, the toxicology results for the clinical trials would also be essential to establish safety. These results should include data related to local tolerance and repeat dose toxicology performed at preclinical settings.

## 7. Conclusions and Future Perspective

In conclusion, a carefully designed delivery system for vaccines, together with the choice of a pertinent administration route, are crucial for both enhancing of immunisation effectiveness and improving vaccine stability, and can help managing dose frequency, patient convenience and logistics for mass immunisation. In essence, viral vectors provide a method to deliver genetic material encoding the pathogen’s antigen directly to the host cell, which when produced in the host mounts an immune response. This capitalizes on the vectors’ specificity in infection and can lead to the production of high concentrations of the target antigen. Viral vectors therefore have the advantage of eliciting strong cellular and humoral immune responses. Although pre-existing immunity to the vector may dampen the response, and the safety of replication-competent vectors is questioned, the potential for their use as vaccine delivery mechanisms is promising. Besides viral vectors, there has been significant progress in developing nonviral vector platforms for vaccine delivery, in recent years. Nanocarrier systems, such as liposomes, virosomes and VLPs have made their way to the market. These systems have been shown to prevent premature antigen release and to prolong antigen presentation for potent immunity against viral diseases. However, the majority of other nanocarriers discussed here remain in the early development and preclinical testing stage. Their ultrasmall size and large surface area can lead to aggregation, and hence, concern over the toxicity and safety of these carriers for clinical use. Therefore, better understanding and knowledge in this regard is essential for developing delivery vehicles with clinical potential.

Advanced delivery systems, like hydrogels and microneedles, have also shown a great potential for localised immunisation. The use of hydrogels, both polymeric and peptide-based, has been shown to be successful strategy for the localised delivery of viral antigens in preclinical studies, thanks to the highly viscous, shear thinning, thixotropic and mucoadhesive properties of these material. These vehicle attributes enable the development of injectable and sprayable formulations capable of forming a viral antigen depot at the site of administration, leading to enhanced activation of APCs, and thus improving both humoral and cellular immune responses over a prolonged duration of action. Hydrogels can act both as vehicles for viral antigens and as immunogenic materials when functionalised with adjuvants. Therefore, we expect to see hydrogel-based viral vaccine formulations for both mucosal (IN) and parenteral (IM, SC and TD) immunisation approved for clinical use in the near future. Microneedles have also been widely studied now, both at preclinical and clinical level, for vaccine delivery purposes. Promising results have been obtained with these prototypes, with comparable efficacy, safety and stability being achieved with MN arrays in comparison with IM vaccine administration. The potential of these systems to facilitate mass vaccination programmes, particularly in low-resource settings, and to promote self-administration of vaccines in contexts where it is not desirable for people to physically go to a healthcare setting for this purpose, such as a pandemic situation, is truly massive and could significantly impact vaccine distribution and coverage in the next few years. Nevertheless, it is still necessary for the scientific and regulatory experts to overcome certain hurdles concerning mass production, standardised characterisation and reproducibility of these devices before they can reach the market and have the expected impact.

Despite significant recent advancements in nanomedicine and biotechnology, there are still a limited number of fully approved nanovaccines against viruses at present. The hybrid systems virosomes and VLPs, which are ‘liposome’ like structures decorated with viral proteins, are by far the most clinically successful nanovaccine products; for example, Epaxal^®^ (HAV), Recombivax HB and Engerix-B (HBV) vaccines, as well as the influenza vaccine Inflexal^®^V, have all been clinically approved and widely used since late-1990s. More recently, the prophylactic HPV vaccines Gardasil^®^, Gardasil-9^®^ and Cervarix^®^, also based on virosome and VLP nanocarriers, were approved, Additionally, a closely related liposome-based Herpes Zoster nanovaccine, Shingrix^®^, was fully approved by the FDA in 2017 for patients older than 50 years. In 2020, the first LNP-based nanovaccines against SARS-CoV-2, BNT162b2 and mRNA-1273, have been granted emergency use authorisation to contain the COVID-19 pandemic and are still under Phase 4 trials to assess the long-term effects of these products. Development of nanovaccine products that can meet the stringent quality, safety and efficacy requirements of regulators is indeed a challenging process, which can be attributed to the complex nature of these multicomponent products. Although nanocarriers have proven to enhance immunisation efficacy of vaccines, safety and stability profiles for both nanocarriers and antigenic elements should be carefully scrutinised in light of the relevant regulators’ guidelines. However, there is a lack of harmonisation for regulations of nanotechnology-enabled products and related advanced technologies/devices from these regulatory bodies, causing uncertainty for product developers and hindering the development and marketing of nanovaccine products. Therefore, most clinically approved nanovaccines were developed with direct involvement of regulators from early development stages to identify and agree on the regulatory requirements for the developed product, which is case-by-case due to the very complex and unique nature of individual nanovaccine products. However, agreement on general regulatory guidelines for the quality, safety and efficacy of nanovaccines, including nanospecific testing considerations, will ensure a smooth approval process for safe and effective products.

## Figures and Tables

**Table 1:** Nanovaccines approved for clinical use by regulators.

Product Name	Developer Company	Target Virus	Nanocarrier System	Viral Antigen Cargo	Marketing Authorisation ^1^
BNT162b2	Pfizer-BioNTech	SARS-CoV-2	LNPs	mRNA encoding SARS-CoV-2 spike glycoprotein	Emergency use authorisation in 2020 by FDA, MHRA & EMA
mRNA-1273	Moderna				
Shingrix^®^	GlaxoSmithKline	Herpes Zoster	Liposomes	Recombinant VZV glycoprotein E	Approved in 2017 for patients >50 years by FDA
Epaxal^®^	Crucell, Berna Biotech (acquired by Johnson & Johnson in 2011)	Hepatitis A	Virosomes & VLPs	Formalin inactivated HAV	Approved in 1993 by EMA Discontinued by Johnson & Johnson in 2011
Recombivax HB	Merk	Hepatitis B		Recombinant HBsAg	Approved in 1986 by FDA
Engerix-B	GlaxoSmithKline				Approved in 2000 by EMA
Inflexal^®^V	Crucell, Berna Biotech (acquired by Johnson & Johnson in 2011)	Influenza H1N1,H3N2 and B		Hemagglutinin and neuraminidase	Approved in 1997 in Switzerland. National authorisation in UK and EU countries Discontinued by Johnson & Johnson in 2011
Gardasil^®^	Merck Sharp & Dohme	Human papillomavirus types 6, 11, 16 and 18		Recombinant L1 proteins of HPV types 6, 11, 16 and 18	Approved in 2006 by EMA
Gardasil-9^®^		Human papillomavirus types 6, 11, 16 18, 31, 33, 45, 52 and 58		Recombinant L1 proteins of HPV types 6, 11, 16 18, 31, 33, 45, 52 and 58	Approved in 2015 by EMA
Cervarix^®^	GlaxoSmithKline	Human papillomavirus types 16 and 18		Recombinant L1 proteins of HPV types 16 and 18	Approved in 2007 for patient ≥9 years by EMA

^1^ Products passed phase 3 clinical trials and got marketing authorisation for use in general population.

**Table 2:** The main elements of the regulatory approval process and their requirements.

Principle Elements	Requirements
Preparation of preclinical materials	Proof of concept testing in animal models Manufacture of clinical material in accordance with cGMP Toxicology investigations in an applicable animal model
Investigational new drug submission	Application for regulatory review
Safety and efficacy testing	Clinical and nonclinical studies
Biologics license application to regulators for final review and licensure	Submission of clinical, nonclinical and manufacturing data
